# Supplementary material for: New myxobacteria of the Myxococcaceae clade produce angiolams with antiparasitic activities
Source: Microbiol Spectr. 2024 Feb 1;12(3):e03689-23. doi: 10.1128/spectrum.03689-23 (PMC10913735; doi:10.1128/spectrum.03689-23)
Supplement: Supplemental material — Supplemental methods and results. [file spectrum.03689-23-s0001.pdf]

Supporting information

New myxobacteria of the *Myxococcaceae* clade produce angiolams with antiparasitic activities

Sebastian Walesch[a,b,c,d], Ronald Garcia[a,b,c,d], Abdelhalim B. Mahmoud[a,b,c,d,e], Fabian Panter[a,b,c,d,f], Sophie Bollenbach[a,b,c,d], Pascal Mäser[g,h], Marcel Kaiser[g,h], Daniel Krug[a,b,c,d], and Rolf Müller[a,b,c,d,f*]

[a] Helmholtz Institute for Pharmaceutical Research Saarland (HIPS), Helmholtz Centre for Infection Research, Saarland University Campus, 66123 Saarbrücken, Germany

[b] Department of Pharmacy, Saarland University, 66123 Saarbrücken, Germany

[c] Helmholtz Centre for Infection research (HZI), Braunschweig, Germany

[d] DZIF - German Center for Infection Research, partner site Hannover-Braunschweig

[e] Faculty of Pharmacy, University of Khartoum, Khartoum, Sudan

[f] Helmholtz International Lab for Anti-Infectives, Campus E8 1, 66123 Saarbrücken, Germany

[g] Parasite Chemotherapy Unit, Swiss Tropical and Public Health Institute, Allschwil, Switzerland

[h] Faculty of Science, University of Basel, Basel, Switzerland

[*] Address correspondence to Rolf Müller, rolf.müller@helmholtz-hips.de.

Table of Contents

1 Materials and methods 2

1.1 Bacterial strains, oligonucleotides, plasmids and cultivation media 2

2 Results 7

2.1 Observation and production of novel angiolam derivatives 7

2.2 Purification and structure elucidation 9

2.2.1 NMR-based comparison of purified angiolam A with authentic angiolam A 9

2.2.2 NMR-based structure elucidation of novel angiolam derivatives 14

2.3 Analysis of angiolam BGC 21

2.3.1 Origin of ethyl-residues in angiolams D and F 28

2.4 Disruption of angiolam BGC by single cross-over inactivation 29

3 NMR spectra employed in angiolam structure elucidation 30

4 References 60

1 Materials and methods

1.1 Bacterial strains, oligonucleotides, plasmids and cultivation media

Table S1: List of strains used in this study

**Strain**	**Function**	**Source**
E. coli HS996	Standard cloning strain	Invitrogen
Pyxidicoccus fallax An d48	Genetically amenable producer of angiolam, used for angBGC knockout	MINS-Lab, GBF[1]
P. fallax An d48-AngKO	Transformant of *P. fallax* An d48 with gene disruption in the angBGC by single cross-over	This study
MCy12716	Producer of angiolam, isolated in “Sample das Saarland” project	This study

MCy12733	Producer of angiolam, isolated in “Sample das Saarland” project	This study

Table S2: List of primers

**Primer name**	**Primer sequence 5’-3’**
48_Ang_Fw	TATAGAATTCGCAGTCCTATCAGGTCCACC
48_Ang_Rv	TATAGAATTCACACGTCGAGGAAGTTGAGG
M13-24F	CCAGGGTTTTCCCAGTCACG
M13-24R	CGGATAACAATTTCACACAGG
48_Test_Fw	GCTATCTGGAGATGGCGCTC
48_Test_Rv	AGCTGGTTGTTGGCGTAGTA

Table S3: List of plasmids

**Name**	**Function**	**Origin**
pCR2.1-TOPO	Cloning vector for single cross-over gene disruptions	Thermo Fisher Scientific
pTOPO-AngKO	pCR2.1-TOPO derivative containg parts of the *angB* gene for the gene disruption of the *angB* gene by single crossover inactivation	This study

Cultivation media

Each medium used in this study was prepared with deionised water. To create a solid medium of any of those listed below, agar (BD) was added to a final concentration of 15 g/L, for soft agar to a final concentration of 7.5 g/L. All media were sterilised by autoclaving at 121 °C, 2 bar for 20 min. For cloning purposes, to select transformants with integrated vector and all further cultivations of these transformants, 50 µg/mL kanamycin was added to the respective media.

LB-medium was used for cultivation of *E. coli*, all other media were used for myxobacteria cultivation.

Table S4: List of culture medium recipes

LB medium		
**Amount**	**Ingredient**	**Supplier**
10 g/L	Tryptone	Roth
5 g/L	Yeast extract	Roth
5 g/L	NaCl	Grüssing
PH adjusted to 7.2 with 1 N KOH.		
**Cy/H medium**		
**Amount**	**Ingredient**	**Supplier**
1.5 g/L	Casitone	BD
1.5 g/L	Yeast extract	BD
4.0 g/L	Soluble starch	Roth
1.0 g/L	Soy flour	Hensel
1.0 g/L	Glucose	Roth
1.0 g/L	$CaCl_2$ x 2 H_2O	Sigma Aldrich
0.5 g/L	$MgSO_4$ x 7 H_2O	Grüssing
11.9 g/L	HEPES	Roth
8.0 mg/L	EDTA-iron*	Sigma Aldrich
0.5 mg/L	Vitamin B_{12}**	Roth
PH adjusted to 7.4 with 10 N KOH.		
**RG5 medium**		
**Amount**	**Ingredient**	**Supplier**
0.5 g/L	Soy peptone	Roth
0.5 g/L	Soytone	BD
2.0 g/L	Soy flour	Hensel
1.0 g/L	Corn steep solids	Sigma Aldrich
0.5 g/L	Yeast extract	BD
8.0 g/L	Soluble starch	Roth

5.0 g/L	Baker's yeast	Wonnemeyer
2.0 g/L	Gluten from wheat	Sigma Aldrich
1.0 g/L	$MgSO_4$ x 7 H_2O	Grüssing
1.0 g/L	$CaCl_2$ x 2 H_2O	Sigma Aldrich
5.95 g/L	HEPES	Roth
PH adjusted to 7.0 with 10 N KOH.		
**VY/2 medium**		
**Amount**	**Ingredient**	**Supplier**
5.0 g/L	Fresh baker's yeast	Wonnemeyer
1.0 g/L	$CaCl_2$ x 2 H_2O	Sigma Aldrich
0.1 mg/L	Vitamin B_{12}**	Roth
PH adjusted to 7.2 with 1 N KOH.		
**YM medium**		
**Amount**	**Ingredient**	**Supplier**
5.0 g/L	Pepton phyton	BD
10.0 g/L	Glucose	Roth
3.0 g/L	Malt extract	BD
3.0 g/L	Yeast extract	BD
1.0 g/L	$MgSO_4$ x 7 H_2O	Grüssing
1.0 g/L	$CaCl_2$ x 2 H_2O	Sigma Aldrich
1 mL/L	0.1 M $K_xH_yPO_4$ solution	Sigma Aldrich
10 mM	Tris x HCl, pH 8.0	Sigma Aldrich
PH adjusted to 7.5 with 10 N KOH.		

* Prepared as stock solution of 8.0 g/L Fe-EDTA complex in deionised water and autoclaved individually. Stock solution is added to medium prior cultivation.

** Prepared as stock solution of 0.5 g/L Vitamin B12 in deionised water, filter sterilised using a 0.22 µm filter and stored at 4 °C. Stock solution is added to medium prior cultivation.

Table S 5: PCR mix for amplification of the *angB* region defined as KO region

4 µL 5 x GC buffer
1 µL DMSO
1 µL Forward primer (10 µM)
1 µL Reverse primer primer (10 µM)
0.4 µL dNTP-mix (10 mM)
0.4 µL gDNA template (~50 ng/ µL)
0.2 µL Phusion DNA Polymerase
12 µL Milli-Q. water

Table S 6: PCR mix to verify integration into the ang BGC by single crossover

4 µL 5 x HF buffer
4 µL DMSO
1 µL gDNA template (~50 ng/ µL)
0.5 µL Forward primer (10 µM)
0.5 µL Reverse primer primer (10 µM)
0.5 µL dNTP-mix (10 mM)
0.2 µL Phusion DNA Polymerase
9.3 µL Milli-Q. water

Table S7: PCR cycler protocol for amplification of KO region

**Step**	**Time [min:s]**	**Temperature [°C]**
**Initial denaturation**	3:00	98
**Cycle, repeat 30 x**	0:20<br>0:30<br>0:30 per kbp to amplify	98<br>68<br>72
**Final elongation**	10:00	72

Table S8: PCR mix to verify integration in ang BGC

**Step**	**Time [min:s]**	**Temperature [°C]**
**Initial denaturation**	3:00	98
**Cycle, repeat 30 x**	0:20<br>0:30<br>0:30 per kbp to amplify	98<br>64<br>72
**Final elongation**	10:00	72

2 Results

2.1 Detection and production of novel angiolam derivatives

Table S 9: Further information on observed angiolam derivatives

Name	Rt [min]	Observed Ion and *m/z* ratio	Sum formula
Angiolam A (**1**)	11.06	$[M-H_2O+H]^+$: 570.379 u	$C_{34}H_{53}NO_7$
Angiolam B (**2**)	11.80	$[M+H]^+$: 586.374 u	$C_{34}H_{51}NO_7$
Angiolam C (**3**)	11.57	$[M-H_2O+H]^+$: 572.395 u	$C_{34}H_{55}NO_7$
Angiolam D_1 (**4a**)	11.61	$[M-H_2O+H]^+$: 584.395 u	$C_{35}H_{55}NO_7$
Angiolam D_2 (**4b**)	11.81	$[M-H_2O+H]^+$: 584.395 u	$C_{35}H_{55}NO_7$
Angiolam E	10.58	$[M+H]^+$: 586.374 u	$C_{34}H_{51}NO_7$
Angiolam F (**5**)	12.14	$[M-H_2O+H]^+$: 598.408 u	$C_{36}H_{57}NO_7$
Angiolam G	10.59	$[M-H_2O+H]^+$: 556.363 u	$C_{33}H_{51}NO_7$

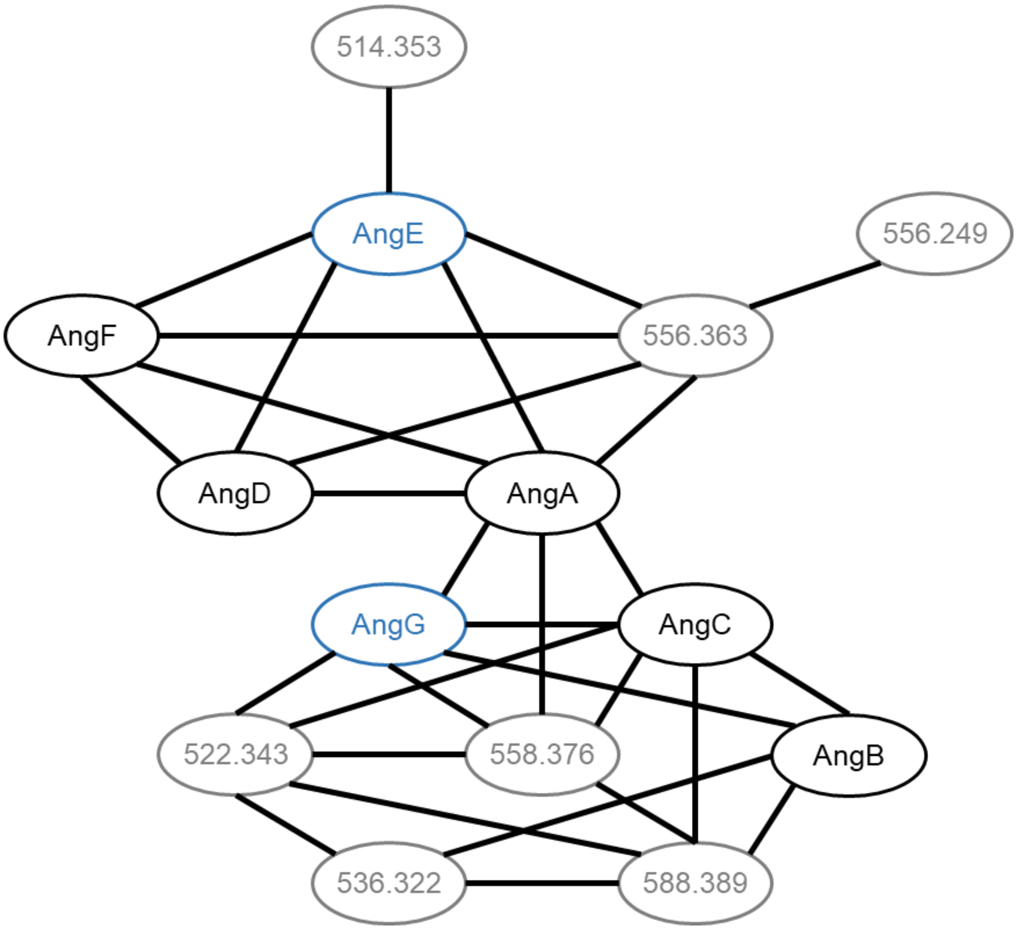

Figure S 1: Feature based molecular network of angiolam derivatives created by GNPS[2,3], observed in crude extracts of Mcy12733 in CyH with and without 2.5 g/L L-valine or RG5. All purified angiolam derivatives are marked black, derivatives observed in CyH and RG5 cultivations are marked blue and derivatives only observed in RG5 cultivations are marked grey.

Table S 10: Production of angiolam derivatives in different media

	Cy/H		Cy/H-Val[c]		RG5	
	Area	Average Peak area	Area	Average Peak area	Area	Average Peak area
**A[a]**	6087406<br>6026506<br>6446253	6186722	1497941<br>1210982<br>1219833	1309585	15847265<br>14044722<br>11793973	13895320
**B[b]**	1654065<br>1450610<br>1538517	1547731	426438<br>429611<br>278645	378231	4251220<br>2444472<br>3377795	3357829
**C[b]**	4212455<br>4133524<br>3906647	4084209	1139368<br>1349834<br>945291	1144831	6846988<br>4413185<br>5494772	5584982
**D[b]**	572186<br>598411<br>537879	569492	3241019<br>3977963<br>2876223	3365068	1276269<br>982698<br>1181980	1146982
**E[b]**	153573<br>134020<br>154004	147199	32198<br>39869<br>26674	32914	515338<br>361496<br>463841	446892
**F[b]**	0<br>0<br>0	0	871836<br>1074687<br>834723	927082	0<br>0<br>0	0
**G[b]**	1060346<br>1091466<br>1018383	1056732	351480<br>502060<br>354269	402603	1242016<br>793880<br>1005443	1013780

[a] Areas were calculated from a 1:40 diluted crude extract.
[b] Areas were calculated from a 1:10 diluted crude extract.
[c] Cy/H medium with added 2.5 g/L L-valine.

Table S 11: Relative production of angiolam derivatives in different media

	Cy/H [%]	Cy/H-Val[a] [%]	RG5 [%]
**A**	100	21	225
**B**	100	24	217
**C**	100	28	137
**D**	100	591	201
**E**	100	22	304
**F**	0	100	0
**G**	100	38	96

[a] Cy/H medium with added 2.5 g/L L-valine.

Relative production levels of angiolam derivatives were normalised to their respective production in Cy/H medium.

2.2 Purification and structure elucidation

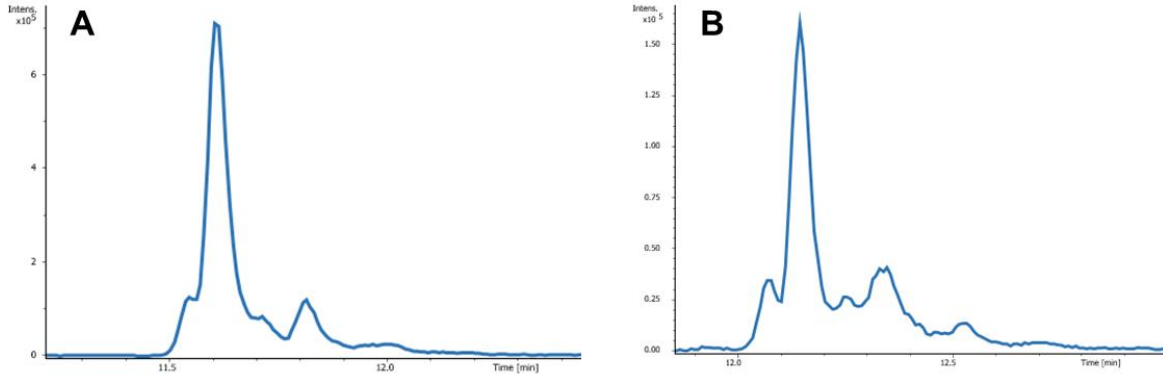

Figure S 2: Multicomponent mixtures of angiolams D (A) and F (B) in crude extracts of Mcy12733 in Cy/H with 2.5 g/L L-valine. Extracted ion chromatograms (EICs) of *m/z* 584.395 (A) and 598.408 (B).

2.2.1 NMR-based comparison of purified angiolam A with authentic angiolam A

As previous structure elucidation of angiolam A (**1**) was performed based on mass-spectrometry, 1D and (selective) NOE-based NMR experiments and X-ray analysis[4], authentic angiolam A was acquired from the Helmholtz Centre for infection research (HZI) for comparison. Evaluation of ^{1}H, ^{13}C and HSQC NMR spectra of authentic and newly purified angiolam A verified that the newly purified natural product was indeed angiolam A (see spectra overlay in Figures S 9 & S 10 and Table S 12 & S 13). Due to organic synthesis of angiolam A, the stereochemistry was verified previously[5]. Furthermore, a new numbering of carbon atoms was introduced, following the biosynthesis of angiolam A (See Figure 3).

Table S 12: ^{1}H-NMR spectroscopic data of authentic angiolam A vs angiolam A from Mcy12733

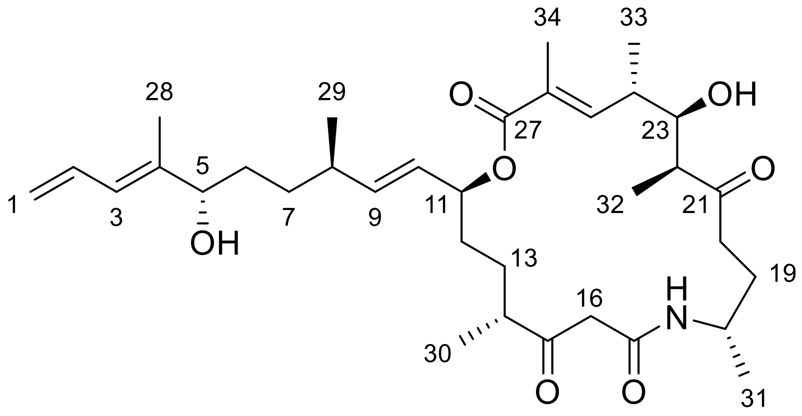

Position	δ_H[a] [ppm] of angiolam A from Mcy12733	δ_H[a] [ppm] of authentic angiolam A	Δ ($\delta_{H\ angiolam\ A\ from\ Mcy12733}$ – $\delta_{H\ authentic\ angiolam\ A}$)
1a	5.20	5.20	0.00
1b	5.11	5.11	0.00
2	6.59	6.59	0.00
3	6.04	6.04	0.00
4	-	-	-
5	4.00	4.00	0.00
6	1.53	1.53	0.00
7a	1.36	1.37	-0.01
7b	1.25	1.25	0.00
8	2.13	2.13	0.00
9	5.53	5.53	0.00
10	5.41	5.41	0.00
11	5.14	5.15	-0.01
12a	1.70	1.71	-0.01
12b	1.50	1.50	0.00
13a	1.83	1.82	0.01
13b	1.32	1.32	0.00
14	2.65	2.65	0.00
15	-	-	-
16a	3.57	3.57	0.00
16b	3.26	3.26	0.00
17	-	-	-
18	4.02	4.02	0.00
19a	1.86	1.85	0.01
19b	1.54	1.53	0.01
20a	2.67	2.67	0.00
20b	2.53	2.53	0.00
21	-	-	-
22	2.64	2.64	0.00
23	3.76	3.76	0.00
24	2.59	2.58	0.01
25	6.49	6.49	0.00
26	-	-	-

27	-	-	-
28	1.74	1.74	0.00
29	0.98	0.99	-0.01
30	1.11	1.11	0.00
31	1.19	1.20	-0.01
32	1.05	1.05	0.00
33	1.14	1.14	0.00
34	1.85	1.85	0.00

[a] Acquired in chloroform-*d* at 500 MHz and calibrated to solvent signal at 7.27 ppm.

Table S 13: ^{13}C-NMR spectroscopic data of authentic angiolam A vs angiolam A from Mcy12733

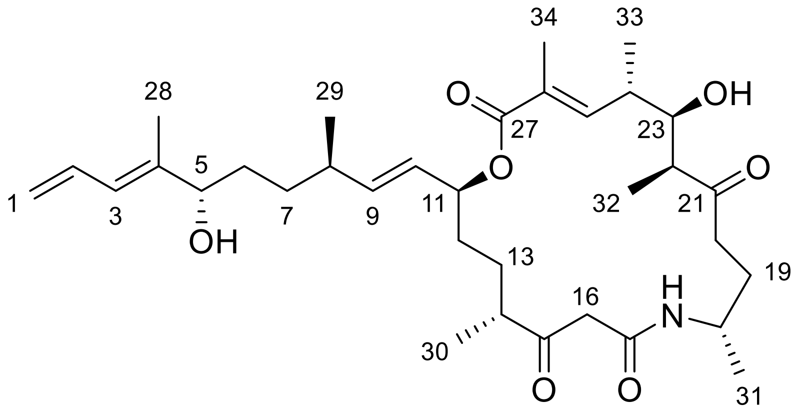

Position	δ_C[a] [ppm] of angiolam A from Mcy12733	δ_C[a] [ppm] of authentic angiolam A	Δ ($\delta_{C\text{ angiolam A from Mcy12733}}$ – $\delta_{C\text{ authentic angiolam A}}$)
1	117.0	117.0	0.0
2	132.7	132.7	0.0
3	125.8	125.8	0.0
4	140.5	140.5	0.0
5	77.2	77.3	-0.1
6	32.8	32.8	0.0
7	32.7	32.7	0.0
8	36.5	36.5	0.0
9	139.3	139.3	0.0
10	126.7	126.7	0.0
11	74.5	74.6	-0.1
12	33.0	33.0	0.0
13	27.7	27.7	0.0
14	47.6	47.6	0.0
15	209.8	209.8	0.0
16	49.2	49.2	0.0
17	165.0	165.0	0.0
18	44.9	44.9	0.0
19	30.5	30.5	0.0
20	38.8	38.8	0.0
21	215.8	215.9	-0.1
22	47.8	47.7	0.1
23	74.0	74.0	0.0

24	36.3	36.3	0.0
25	142.4	142.4	0.0
26	128.7	128.7	0.0
27	167.6	167.7	-0.1
28	12.1	12.1	0.0
29	20.4	20.4	0.0
30	16.0	16.0	0.0
31	21.8	21.8	0.0
32	8.7	8.7	0.0
33	16.7	16.7	0.0
34	12.6	12.6	0.0

[a] Acquired in chloroform-*d* at 125 MHz and calibrated to solvent signal at 77.0 ppm.

Although the identity of newly purified angiolam A (**1**) was verified by NMR comparisons with authentic angiolam A, a full set of ^{1}H, ^{13}C, COSY, HSQC, and HMBC-NMR spectra was recorded for a NMR-based structure elucidation of angiolam A (**1**), purified from MCy12733. COSY-correlations are indicated in bold, relevant HMBC correlations as arrows (Table S 14).

Table S 14: NMR Spectroscopic data for angiolam A (1)

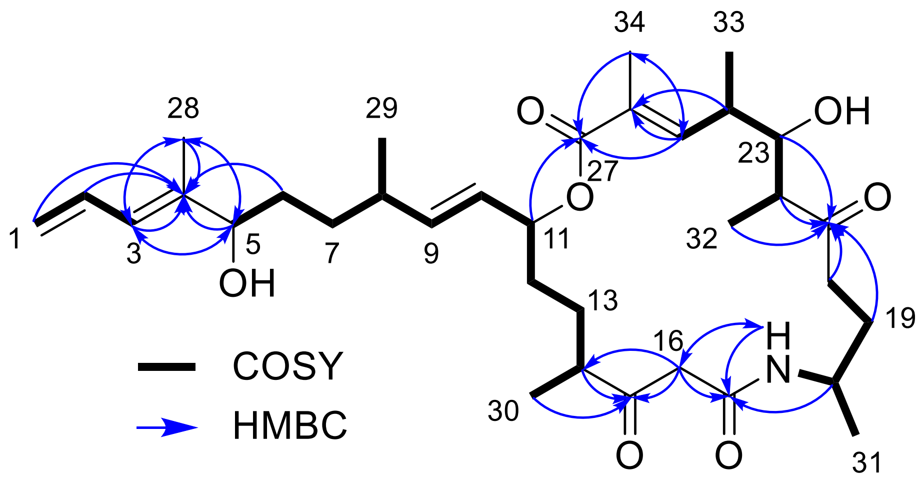

Position	δ_C[a] [ppm]	Type	δ_H[b] [ppm]	H Multiplicity (*J* [Hz])	COSY[c]	HMBC[d]
1a	117.0	CH_2	5.20	m	1b, 2, 3, 28	2, 3
1b			5.11	m	1a, 2, 3, 28	2, 3, 4
2	132.7	CH	6.59	m	1a, 1b 3	1, 3, 4, 5
3	125.8	CH	6.04	m	1a, 1b, 2, 5, 28	1, 2, 4, 5, 6, 28
4	140.5	C	-	-	-	-
5	77.2	CH	4.00	m	3, 6, 28	3, 4, 7, 28

6	32.8	CH_2	1.53	m	5, 7a, 7b	4, 5, 7, 8
7a	32.7	CH_2	1.36	m	6, 7b, 8	5, 6, 8, 9, 29
7b			1.25	m	6, 7a, 8	5, 6, 8, 9, 29
8	36.5	CH	2.13	m	7a, 7b, 9, 10, 29	6, 7, 9, 10, 29
9	139.3	CH	5.53	dd (15.5, 7.9)	8, 10, 11, 29	7, 8, 10, 11, 29
10	126.7	CH	5.41	m	8, 9, 11	8, 9, 11, 12, 29
11	74.5	CH	5.14	m	9, 10, 12a, 12b	9, 10, 12, 13, 27
12a	33.0	CH_2	1.70	m	11, 12b, 13a, 13b	10, 11, 13, 14
12b			1.50	m	11, 12a, 13a, 13b	10, 11, 13, 14
13a	27.7	CH_2	1.83	m	12a, 12b, 13b, 14	11, 12, 14, 15, 30
13b			1.32	m	12a, 12b, 13a, 14	11, 12, 15, 30
14	47.6	CH	2.65	m	13a, 13b, 30	12, 13, 15, 30
15	209.8	C	-	-	-	-
16a	49.2	CH_2	3.57	br s	16b	14, 15, 17, 18
16b			3.26	m	16a	15, 17
17	165.0	C	-	-	-	-
18	44.9	CH	4.02	m	19a, 19b, 31, NH	17, 19, 20, 31
19a	30.5	CH_2	1.86	m	18, 19b, 20a, 20b	18, 20, 21, 31
19b			1.54	m	18, 19a, 20a, 20b	18, 20, 21, 31
20a	38.8	CH_2	2.67	m	19a, 19b, 20b	18, 19, 21
20b			2.53	m	19a, 19b, 20a	18, 19, 21
21	215.8	C	-	-	-	-
22	47.8	CH	2.64	m	23, 32	21, 23, 24, 32
23	74.0	CH	3.76	m	22, 24, 33	21, 24, 25, 32, 33
24	36.3	CH	2.59	m	23, 25, 33	22, 23, 25, 26, 33
25	142.4	CH	6.49	dd (10.1, 1.4)	24, 34	23, 24, 26, 27, 33, 34
26	128.7	C	-	-	-	-
27	167.6	C	-	-	-	-
28	12.1	CH_3	1.74	d (0.8)	1a, 1b, 3, 5	1, 2, 3, 4, 5
29	20.4	CH_3	0.98	m	8, 9	7, 8, 9
30	16.0	CH_3	1.11	d (7.0)	14	13, 14, 15
31	21.8	CH_3	1.19	d (6.6)	18	18, 19, 20
32	8.7	CH_3	1.05	d (7.1)	22	21, 22, 23
33	16.7	CH_3	1.14	d (6.6)	23, 24	23, 24, 25
34	12.6	CH_3	1.85	M	25	23, 24, 25, 26, 27
NH	-	NH	6.25	br d (9.2)	18	16, 17, 18, 19, 31

[a] Acquired in chloroform-*d* at 125 MHz and calibrated to solvent signal at 77.0 ppm.
[b] Acquired in chloroform-*d* at 500 MHz and calibrated to solvent signal at 7.27 ppm.
[c] Proton showing COSY correlations to indicated proton.
[d] Proton showing HMBC correlations to indicated carbon.

2.2.2 NMR-based structure elucidation of novel angiolam derivatives

The structures of the novel angiolam derivatives B (**2**), C (**3**), D_1 (**4a**), D_2 (**4b**) and F (**5**) were elucidated based on ^{1}H, ^{13}C, COSY, HSQC, and HMBC NMR spectra. As all angiolam derivatives are produced by the same biosynthetic machinery, they display the same stereochemistry. Correlations that were used to display the changes of the novel angiolam derivatives compared to angiolam A (**1**) are shown in a bold line for COSY and as arrows for HMBC.

Table S 15: NMR Spectroscopic data for angiolam B (2)

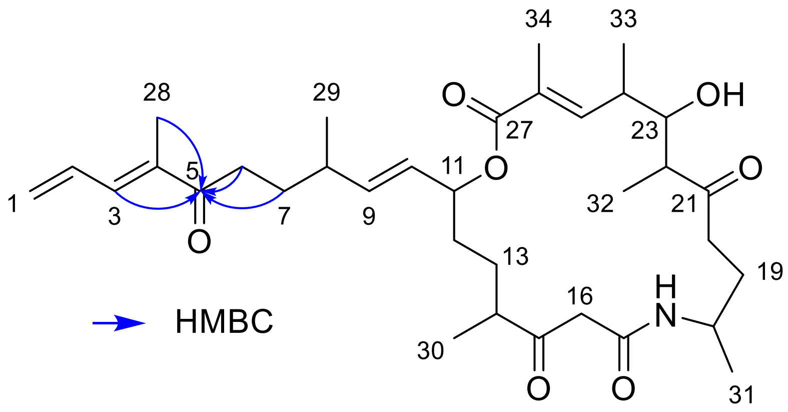

Position	δ_C[a] [ppm]	Type	δ_H[b] [ppm]	H Multiplicity (*J* [Hz])	COSY[c]	HMBC[d]
1a	124.5	CH_2	5.63	br d (18.3)	1b, 2, 3, 28	2, 3, 4
1b	124.5	CH_2	5.50	d (10.1)	1a, 2, 3, 28	3, 5
2	132.9	CH	6.74	dt (16.8, 10.5, 10.5)	1a, 1b, 3	3, 4
3	138.0	CH	7.02	br d (11.0)	1a, 1b, 2, 28	1, 2, 4, 5, 28
4	136.6	C	-	-	-	-
5	202.2	C	-	-	-	-
6a	35.1	CH_2	2.72	m	6b, 7a	5, 7, 8
6b			2.62	m	6a, 7a	5, 7, 8
7a	31.4	CH_2	1.68	m	6a, 6b, 7b, 8	5, 6, 8, 9, 29
7b			1.57	m	7a, 8	5, 6, 8, 9, 29
8	36.2	CH	2.16	m	7a, 7b, 9, 29	6, 7, 9, 10, 29
9	139.4	CH	5.58	m	8, 10	7, 8, 10 ,11, 29
10	127.5	CH	5.44	m	9, 11	8, 9, 11, 29
11	75.1	CH	5.10	m	10, 12a, 12b	9, 10, 27
12a	33.2	CH_2	1.67	m	11, 12b, 13a, 13b	10, 11, 13, 14
12b			1.51	m	11, 12a, 13a, 13b	10, 11, 13, 14
13a	28.0	CH_2	1.81	m	12a, 12b, 13b, 14	11, 12, 14, 15, 30
13b			1.33	m	12a, 12b, 13a, 14	11, 12, 14, 15, 30
14	47.5	CH	2.66	m	13a, 13b, 30	12, 13, 15, 30
15	209.7	C	-	-	-	-
16a	49.3	CH_2	3.56	d (15.9)	16b	14, 15, 17, 18
16b			3.27	d (15.9)	16a	15, 17

17	165.0	C	-	-	-	-
18	45.0	CH	4.02	m	19a, 19b, 31, NH	17, 19, 20, 31
19a	30.5	CH_2	1.85	m	18, 20a, 20b	18, 20, 21, 31
19b			1.54	m	18, 20a, 20b	18, 20, 21, 31
20a	38.7	CH_2	2.73	m	19a, 19b, 20b	18, 19, 21
20b			2.54	m	19a, 19b, 20a	18, 19, 21
21	215.9	C	-	-	-	-
22	47.8	CH	2.66	m	23, 32	20, 21, 23, 32
23	73.9	CH	3.78	br d (10.0)	22, 24	21, 24, 25, 32, 33
24	36.3	CH	2.59	m	23, 25, 33	22, 23, 25, 26, 33
25	142.5	CH	6.52	dd (10.2, 1.3)	24, 34	23, 24, 26, 27, 33, 34
26	128.6	C	-	-	-	-
27	167.8	C	-	-	-	-
28	11.7	CH_3	1.89	s	1a, 1b, 3	1, 2, 3, 4, 5
29	20.4	CH_3	1.02	d (6.7)	8	7, 8, 9
30	16.3	CH_3	1.12	d (7.1)	14	13, 14, 15
31	21.8	CH_3	1.20	d (6.6)	18	18, 19, 20
32	8.8	CH_3	1.06	d (7.2)	22	21, 22, 23
33	16.7	CH_3	1.14	d (6.6)	24	23, 24, 25
34	12.7	CH_3	1.84	d (1.1)	25	23, 24, 25, 26, 27, 33
NH	-	NH	6.17	br d (9.0)	18	17, 18, 19, 31

[a] Acquired in chloroform-*d* at 125 MHz and calibrated to solvent signal at 77.0 ppm.
[b] Acquired in chloroform-*d* at 500 MHz and calibrated to solvent signal at 7.27 ppm.
[c] Proton showing COSY correlations to indicated proton.
[d] Proton showing HMBC correlations to indicated carbon.

Table S 16: NMR Spectroscopic data for angiolam C (3)

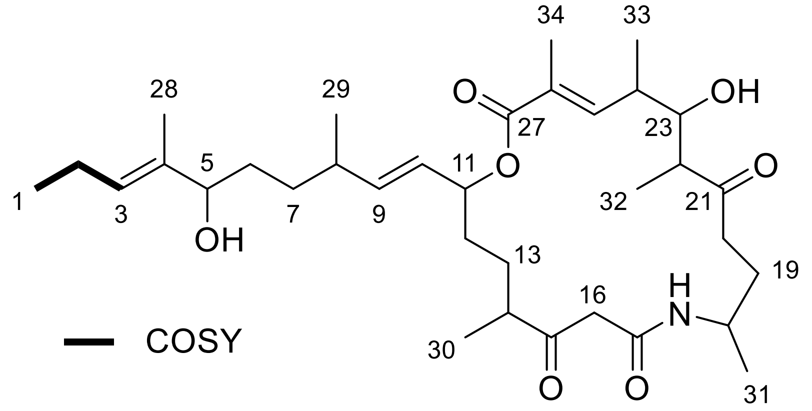

Position	δ_C[a] [ppm]	Type	δ_H[b] [ppm]	H Multiplicity (*J* [Hz])	COSY[c]	HMBC[d]
1	14.1	CH_3	0.97	t (7.5, 7.5)	2, 3	2, 3
2	20.8	CH_2	2.03	m	1, 3	1, 4
3	128.4	CH	5.37	m	1, 2, 5, 28	1, 2, 5, 28
4	136.4	C	-	-	-	-
5	77.9	CH	3.93	br t (6.7, 6.7)	3, 6	3, 4, 7
6	32.9	CH_2	1.52	m	5, 7a, 7b	4, 5, 7, 8
7a	32.7	CH_2	1.35	m	6, 7b, 8	5, 6, 8, 9, 29

7b			1.22	m	6, 7a, 8	5, 9, 29
8	36.5	CH	2.13	dt (13.8, 6.9, 6.9)	7a, 7b, 9, 29	6, 9, 10, 29
9	139.5	CH	5.55	dd (15.4, 7.7)	8, 10, 29	6, 8, 10, 11, 29
10	126.6	CH	5.41	dd (15.4, 6.9)	9, 11	8, 11, 12
11	74.6	CH	5.16	m	10, 12a, 12b	9, 10, 12, 13, 27
12a	33.1	CH_2	1.72	m	11, 12b, 13a, 13b	13, 14
12b			1.51	m	11, 12a, 13a, 13b	11, 13, 14
13a	27.8	CH_2	1.81	m	12a, 12b, 13b, 14	12, 14, 30
13b			1.33	m	12a, 12b, 13a, 14	11, 12, 14, 15, 30
14	47.8	CH	2.67	m	13a, 13b, 30	12, 13, 15, 30
15	209.8	C	-	-	-	-
16a	49.2	CH_2	3.57	d (15.8)	16b	15, 17, 18
16b			3.26	d (15.8)	16a	15, 17
17	165.0	C	-	-	-	-
18	45.0	CH	4.02	m	19a, 19b, 31, NH	17, 19, 31
19a	30.6	CH_2	1.84	m	18, 19b, 20a, 20b	20
19b			1.54	m	18, 19a, 20a, 20b	18, 20
20a	38.8	CH_2	2.68	m	19a, 19b, 20b	18, 19, 21
20b			2.54	m	19a, 19b, 20a	18, 19, 21
21	215.8	C	-	-	-	-
22	47.6	CH	2.64	m	32	20, 21, 23, 24, 32
23	74.1	CH	3.76	br d (9.8)	24	21, 24, 25, 32, 33
24	36.3	CH	2.59	m	23, 25, 33	22, 23, 25, 26, 33
25	142.4	CH	6.49	br d (10.1)	24, 34	23, 24, 26, 27, 33, 34
26	128.7	C	-	-	-	-
27	167.7	C	-	-	-	-
28	11.1	CH_3	1.59	s	3	1, 2, 3, 4, 5
29	20.4	CH_3	0.99	d (6.5)	8, 9	6, 8, 9
30	16.0	CH_3	1.12	br d (7.0)	14	13, 14, 15
31	21.8	CH_3	1.20	br d (6.5)	18, NH	18, 19
32	8.8	CH_3	1.06	d (7.0)	22	21, 22, 23
33	16.7	CH_3	1.15	br d (6.7)	24	23, 24, 25
34	12.6	CH_3	1.86	m	25	24, 25, 26, 27
NH	-	NH	6.25	br d (9.1)	18, 31	17, 18

[a] Acquired in chloroform-*d* at 125 MHz and calibrated to solvent signal at 77.0 ppm.
[b] Acquired in chloroform-*d* at 500 MHz and calibrated to solvent signal at 7.27 ppm.
[c] Proton showing COSY correlations to indicated proton.
[d] Proton showing HMBC correlations to indicated carbon.

Table S 17: NMR Spectroscopic data for angiolam D_1 (4a)

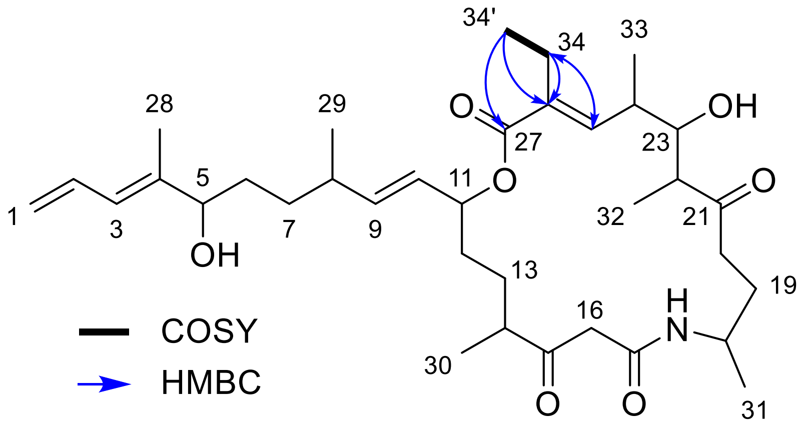

Position	δ_C[a] [ppm]	Type	δ_H[b] [ppm]	H Multiplicity (*J* [Hz])	COSY[c]	HMBC[d]
1a	116.9	CH_2	5.20	m	1b, 2	2, 3, 4
1b			5.11	m	1a, 2	3
2	132.7	CH	6.59	dt (16.8, 10.5, 10.5)	1a, 1b, 3	3, 4
3	125.8	CH	6.04	ddd (11.3, 1.8, 0.8)	2, 28	1, 2, 4, 5, 28
4	140.5	C	-	-	-	-
5	77.3	CH	4.00	m	6	3, 4, 6, 28
6	32.8	CH_2	1.54	m	5, 7a, 7b	4, 5, 7, 8
7a	32.7	CH_2	1.38	m	6, 7b, 8	5, 6, 8, 9, 29
7b			1.27	m	6, 7a, 8	5, 6, 8, 9, 29
8	36.5	CH	2.14	m	7a, 7b, 9, 29	6, 9, 10, 29
9	139.3	CH	5.53	m	8, 10	7, 8, 10, 11, 29
10	126.7	CH	5.42	m	9, 11	8, 9, 11, 12
11	74.5	CH	5.17	m	10, 12a, 12b	9, 10, 13, 27
12a	33.0	CH_2	1.71	u	11, 12b, 13a, 13b	10, 11, 13, 14
12b			1.49	m	11, 12a, 13a, 13b	10, 13, 14
13a	27.7	CH_2	1.83	m	12a, 12b, 13b, 14	
13b			1.33	m	12a, 12b, 13a, 14	11, 14,15, 30
14	47.7	CH	2.66	m	13a, 13b, 30	12, 13, 15, 30
15	209.9	C	-	-	-	-
16a	49.2	CH_2	3.58	m	16b	15, 17, 18
16b			3.25	m	16a	15, 17
17	165.0	C	-	-	-	-
18	44.9	CH	4.03	m	19b, 31, NH	17, 19, 31
19a	30.5	CH_2	1.83	m	20a, 20b	18, 20, 21, 31
19b			1.55	m	18, 20a, 20b	18, 20, 21, 31
20a	38.8	CH_2	2.63	m	19a, 19b, 20b	18, 19, 21
20b			2.53	m	19a, 19b, 20a	18, 19, 21
21	215.8	C	-	-	-	-
22	47.6	CH	2.65	m	23, 32	20, 21, 32
23	74.2	CH	3.76	m	22, 24	21, 25, 32, 33
24	36.0	CH	2.60	m	23, 25, 33	22, 23, 25, 26, 33
25	141.3	CH	6.39	br d (10.2)	24	23, 24, 26, 27, 33, 34
26	135.1	C	-	-	-	-

27	167.4	C	-	-	-	-
28	12.1	CH_3	1.75	d (1.2)	3	2, 3, 4, 5
29	20.4	CH_3	0.98	d (6.7)	8	7, 8, 9
30	15.8	CH_3	1.11	dd (7.0, 1.6)	14	13, 14, 15
31	21.8	CH_3	1.20	dd (6.6, 3.1)	18	18, 19
32	8.8	CH_3	1.05	dd (7.2, 1.8)	22	21, 22, 23
33	17.4	CH_3	1.16	d (6.6)	24	23, 24, 25
34a	20.6	CH_2	2.35	m	34b, 34'	25, 26, 27, 34'
34b			2.29	m	34a, 34'	25, 26, 27, 34'
34'	14.0	CH_3	1.01	m	34a, 34b	26, 34
NH	-	NH	6.29	br d (9.2)	18	17, 18

[a] Acquired in chloroform-*d* at 175 MHz and calibrated to solvent signal at 77.0 ppm.
[b] Acquired in chloroform-*d* at 700 MHz and calibrated to solvent signal at 7.27 ppm.
[c] Proton showing COSY correlations to indicated proton.
[d] Proton showing HMBC correlations to indicated carbon.

Table S 18: NMR Spectroscopic data for angiolam D_2 (4b)

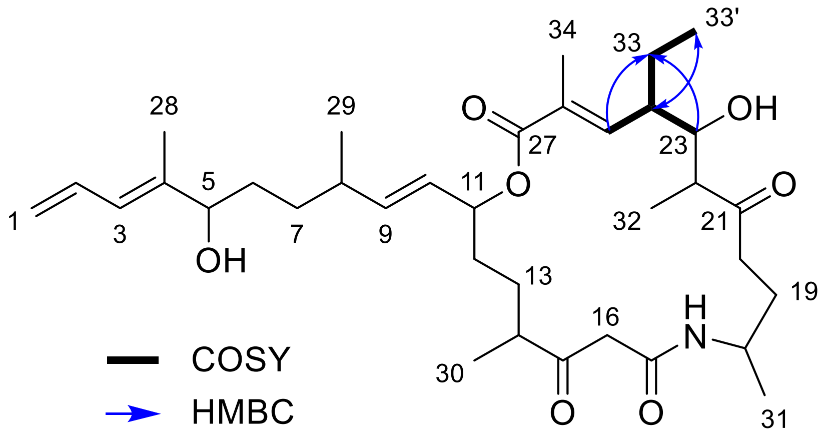

Position	δ_C[a] [ppm]	Type	δ_H[b] [ppm]	H Multiplicity (*J* [Hz])	COSY[c]	HMBC[d]
1a	116.9	CH_2	5.20	m	1b, 2	2, 3, 4
1b			5.11	m	1a, 2	3
2	132.7	CH	6.59	dt (16.8, 10.5, 10.5)	1a, 1b, 3	3, 4
3	125.8	CH	6.04	ddd (11.3, 1.8, 0.8)	2, 28	1, 2, 4, 5, 28
4	140.5	C	-	-	-	-
5	77.3	CH	4.00	m	6	3, 4, 6, 28
6	32.8	CH_2	1.54	m	5, 7a, 7b	4, 5, 7, 8
7a	32.7	CH_2	1.38	m	6, 7b, 8	5, 6, 8, 9, 29
7b			1.27	m	6, 7a, 8	5, 6, 8, 9, 29
8	36.5	CH	2.14	m	7a, 7b, 9, 29	6, 9, 10, 29
9	139.3	CH	5.53	m	8, 10	7, 8, 10, 11, 29
10	126.7	CH	5.42	m	9, 11	8, 9, 11, 12
11	74.5	CH	5.17	m	10, 12a, 12b	9, 10, 13, 27
12a	33.0	CH_2	1.71	m	11, 12b, 13a, 13b	10, 11, 13, 14
12b			1.49	m	11, 12a, 13a, 13b	10, 13, 14
13a	27.7	CH_2	1.83	m	12a, 12b, 13b, 14	
13b			1.33	m	12a, 12b, 13a, 14	11, 14,15, 30

14	47.7	CH	2.66	m	13a, 13b, 30	12, 13, 15, 30
15	209.9	C	-	-	-	-
16a	49.2	CH_2	3.58	m	16b	15, 17, 18
16b			3.25	m	16a	15, 17
17	165.0	C	-	-	-	-
18	44.9	CH	4.03	m	19b, 31, NH	17, 19, 31
19a	30.5	CH_2	1.83	m	20a, 20b	18, 20, 21, 31
19b			1.55	m	18, 20a, 20b	18, 20, 21, 31
20a	38.8	CH_2	2.63	m	19a, 19b, 20b	18, 19, 21
20b			2.53	m	19a, 19b, 20a	18, 19, 21
21	215.8	C	-	-	-	-
22	48.0	CH	2.57	m	23, 32	21
23	72.4	CH	3.84	dd (9.8, 1.3)	22, 24	21, 24, 25, 33
24	43.2	CH	2.46	m	23, 25, 33a, 33b	23, 25, 26, 33, 33'
25	141.4	CH	6.41	dd (10.8, 1.4)	24, 34	23, 24, 26, 27, 33, 34
26	130.1	C	-	-	-	-
27	167.5	C	-	-	-	-
28	12.1	CH_3	1.75	d (1.2)	3	2, 3, 4, 5
29	20.4	CH_3	0.98	d (6.7)	8	7, 8, 9
30	15.8	CH_3	1.11	dd (7.0, 1.6)	14	13, 14, 15
31	21.8	CH_3	1.20	dd (6.6, 3.1)	18	18, 19
32	8.9	CH_3	1.05	m	22	21, 22, 23
33a	24.2	CH_2	2.00	m	24, 33b, 33'	33'
33b			1.33	m	24, 33a, 33'	24, 33'
33'	11.2	CH_3	0.86	t (7.5, 7.5)	33a, 33b	24, 33
34	13.2	CH_3	1.87	d (1.4)	25	23, 24, 25, 26, 27, 33
NH		NH	6.29	br d (9.2)	18	17, 18

[a] Acquired in chloroform-*d* at 175 MHz and calibrated to solvent signal at 77.0 ppm.
[b] Acquired in chloroform-*d* at 700 MHz and calibrated to solvent signal at 7.27 ppm.
[c] Proton showing COSY correlations to indicated proton.
[d] Proton showing HMBC correlations to indicated carbon.

Table S 19: NMR Spectroscopic data for angiolam F (5)

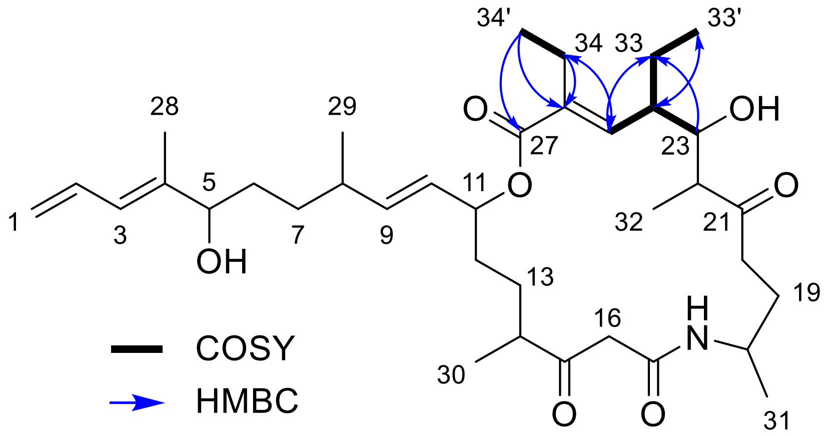

Position	δ_C[a] [ppm]	Type	δ_H[b] [ppm]	H Multiplicity (J [Hz])	COSY[c]	HMBC[d]
1a	117.0	CH_2	5.20	m	1b, 2, 3	2, 3
1b			5.11	br d (10.0)	1a, 2, 3	3
2	132.7	CH	6.59	dt (16.8, 10.6, 10.6)	1a, 1b, 3	3, 4
3	125.8	CH	6.04	br d (11.1)	1a, 1b, 2, 5, 28	1, 2, 5, 28
4	140.5	C	-	-	-	-
5	77.3	CH	4.00	m	3, 6	3, 4, 7, 28
6	32.9	CH_2	1.53	m	5, 7a, 7b	4, 5, 7, 8
7a	32.8	CH_2	1.37	m	6, 8	5, 8, 9
7b			1.24	m	6, 8	8, 9
8	36.7	CH	2.14	dt (13.5, 6.9, 6.9)	7a, 7b, 9, 29	6, 9, 10, 29
9	139.2	CH	5.52	m	8, 10	7, 8, 10, 11, 29
10	126.7	CH	5.43	dd (15.5, 6.4)	9, 11	8, 9, 11, 12, 29
11	74.1	CH	5.22	m	10, 12a, 12b	9, 13, 27
12a	32.9	CH_2	1.74	m	11, 12b, 13a, 13b	10, 11, 14
12b			1.50	m	11, 12a, 13a, 13b	
13a	27.6	CH_2	1.84	m	12a, 12b, 14	
13b			1.32	m	12a, 12b, 14	14, 15, 30
14	47.7	CH	2.65	m	13a, 13b, 30	12, 13, 15, 30
15	209.9	C	-	-	-	-
16a	49.0	CH_2	3.59	br d (15.7)	16b	15, 17
16b			3.24	d (15.7)	16a	15, 17
17	164.9	C	-	-	-	-
18	44.9	CH	4.03	m	19a, 19b, 31	17, 31
19a	30.5	CH_2	1.85	m	18, 19b, 20a, 20b	18, 20, 21, 31
19b			1.52	m	18, 19a, 20a, 20b	18, 20, 21, 31
20a	38.8	CH_2	2.67	m	19a, 19b	18, 19, 21
20b			2.55	m	19a, 19b	18, 19, 21
21	216.0	C	-	-	-	-
22	47.7	CH	2.60	m	23, 32	21, 32
23	72.4	CH	3.82	br d (9.8)	22, 24	21, 24, 25, 32, 33
24	42.8	CH	2.45	ddd (19.7, 9.6, 2.9)	23, 25, 33	23, 25, 26, 33, 33'
25	139.9	CH	6.26	d (10.8)	24	23, 24, 26, 27, 33, 34, 34'

26	136.6	C	-	-	-	-
27	167.3	C	-	-	-	-
28	12.1	CH_3	1.75	m	3	3, 4, 5
29	20.6	CH_3	0.98	m	8	7, 8, 9
30	15.7	CH_3	1.11	d (6.9)	14	12, 13, 14, 15
31	21.9	CH_3	1.20	d (6.6)	18	18, 19
32	8.8	CH_3	1.06	br d (7.1)	22	21, 23
33	24.2	CH_2	1.98	m	24, 33'	23, 24, 25
33'	11.2	CH_3	0.86	m	33	24, 33
34	20.7	CH_2	2.34	m	34'	25, 26, 27, 34'
34'	13.6	CH3	0.99	m	34	26, 34
NH	-	NH	6.32	br d (9.3)	-	17, 18

[a] Acquired in chloroform-*d* at 125 MHz and calibrated to solvent signal at 77.0 ppm.
[b] Acquired in chloroform-*d* at 500 MHz and calibrated to solvent signal at 7.27 ppm.
[c] Proton showing COSY correlations to indicated proton.
[d] Proton showing HMBC correlations to indicated carbon.

2.3 Analysis of angiolam BGC

Based on *in silico* analyses of the genomes of MCy12716 and MCy12733 with antiSMASH[6], the ang BGC could be identified. All coding sequences surrounding the genetic locus encoding th angiolam biosynthetic machinery in both strains were extracted from the MCy12733 (ctg39_13-ctg39_27) genome sequence, translated and searched with the blastp algorithm against the RefSeq non-redundant protein sequence database at NCBI. Furthermore, the gene putatively encoding a fused isobutyryl-CoA mutase (ctg31_31) was treated accordingly.

Table S 20: Blastp results of the CDS regions in the ang BGC

CDS Name	Length [AA]	Closest homologue [Organism of origin]	Identity [%] and query coverage [%]	Accession Nr.
ang1	393	acetyl-CoA C-acetyltransferase [Pyxidicoccus caerfyrddinensis]	98.2; 100	WP_163996527.1
ang2	232	CoA transferase subunit A [Pyxidicoccus caerfyrddinensis]	98.7; 100	WP_163996526.1
ang3	218	CoA transferase subunit B [Pyxidicoccus caerfyrddinensis]	100,0; 100	WP_163996525.1
ang4	131	TIGR02266 family protein [Pyxidicoccus caerfyrddinensis]	98.5; 100	WP_163996524.1
ang5	431	class I SAM-dependent RNA methyltransferase [Pyxidicoccus caerfyrddinensis]	97.9; 100	WP_163996523.1
ang6	465	VWA domain-containing protein [Pyxidicoccus caerfyrddinensis]	100.0; 100	WP_163996522.1

angA	2672	SDR family NAD(P)-dependent oxidoreductase [Pyxidicoccus fallax]	82.1; 99	NMO18055.1
angB	3652	type I polyketide synthase [Pyxidicoccus fallax]	85.4; 99	WP_169347329.1
angC	6523	type I polyketide synthase [Pyxidicoccus fallax]	84.1; 96	WP_169345687.1
angD	5062	amino acid adenylation domain-containing protein [Pyxidicoccus fallax]	85.3; 100	NMO16395.1
angE	3855	type I polyketide synthase [Pyxidicoccus fallax]	83.4; 99	WP_169345685.1
angF	386	acyl-CoA dehydrogenase family protein [Pyxidicoccus fallax]	89.4; 99	WP_169345684.1
ang7	397	efflux RND transporter periplasmic adaptor subunit [Pyxidicoccus caerfyrddinensis]	97.7; 100	WP_163996521.1
ang8	1043	efflux RND transporter permease subunit [Pyxidicoccus caerfyrddinensis]	98.6; 100	WP_163996520.1
ctg31_31	1155	methylmalonyl-CoA mutase family protein [Pyxidicoccus caerfyrddinensis]	98.9; 100	WP_163996481.1

As all observed CDS regions belong to the genus of *Pyxidicoccus* and share high similarities with the query sequences, the first non-*Pyxidicoccus* hits were also retrieved.

Table S 21: Blastp results of the CDS regions in the *ang* BGC without the genus *Pyxidicoccus*

CDS Name	Length [AA]	Closest homologue without *Pyxidicoccus* strains [Organism of origin]	Identity [%] and query coverage [%]	Accession Nr.
ang1	393	acetyl-CoA C-acetyltransferase [Myxococcus sp. RHSTA-1-4]	93.4; 100	WP_223754820.1
ang2	232	CoA transferase subunit A [Myxococcus sp. RHSTA-1-4]	97.0; 100	WP_223754821.1
ang3	218	CoA transferase subunit B [Myxococcus sp. RHSTA-1-4]	95.9; 100	WP_223754822.1
ang4	131	TIGR02266 family protein [Myxococcus sp. RHSTA-1-4]	88.6; 100	WP_223754823.1
ang5	431	class I SAM-dependent RNA methyltransferase [Myxococcus sp. RHSTA-1-4]	94.9; 100	WP_223754824.1
ang6	465	VWA domain-containing protein [Myxococcus sp. XM-1-1-1]	92.7; 100	WP_223748567.1
angA	2672	type I polyketide synthase [Polyangium spumosum DSM14734]	42.7; 97	WP_153821595.1
angB	3652	epoD [Sorangium cellulosum SMP44]	52.1; 99	AAF62883.1
angC	6523	epoD [Sorangium cellulosum SMP44]	46.8; 99	AAF62883.1

angD	5062	epoD [Sorangium cellulosum SMP44]	61.9; 79	AAF62883.1
angE	3855	polyketide synthase [Sorangium cellulosum So0157-2]	69.1; 84	ACB46196.1
angF	386	acyl-CoA/acyl-ACP dehydrogenase [Enhygromyxa salina]	63.7; 99	WP_052559235.1
ang7	397	efflux RND transporter periplasmic adaptor subunit [Myxococcus sp. RHSTA-1-4]	77.6; 100	WP_223755293.1
ang8	1043	efflux RND transporter permease subunit [Myxococcus sp. RHSTA-1-4]	88.3; 100	WP_223755292.1
ctg31_31	1155	methylmalonyl-CoA mutase family protein [Corallococcus sp. CA053C]	93.3; 100	WP_120611036.1

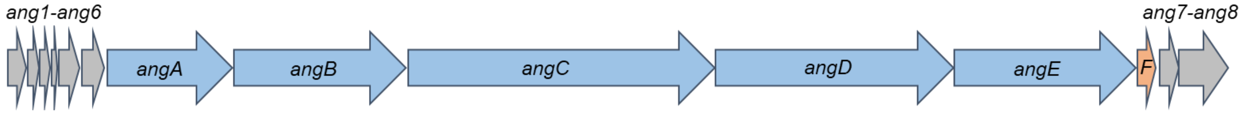

Figure S 3: Gene cluster arrow figure of the extended *ang* BGC. The core biosynthetic genes are displayed as blue (PKS-NRPS genes; *angA-angE*) or orange arrows (putative acyl-CoA/acyl-ACP dehydrogenase; *angF*), the additional genes (*ang1-ang6* & *ang7-ang8*) in the same open reading frame are displayed as grey arrows. Arrow lenghts display the relative lenghts of all genes.

Table S 22: Substrate specificity of angiolam BGC AT domains

	**Main substrate**	**AntiSMASH[6]**	**Fingerprint[7]**	**RIDVV-motif[8]**
L	Methylmanonyl-CoA	Methylmanonyl-CoA[a]	Methylmanonyl-CoA	No
M1	Methylmanonyl-CoA	Methylmanonyl-CoA[a]	Methylmanonyl-CoA	No
M2	Malonyl-CoA	Malonyl-CoA	Malonyl-CoA	
M3	Methylmanonyl-CoA	Methylmanonyl-CoA[a]	Methylmanonyl-CoA	Yes
M4	Malonyl-CoA	Malonyl-CoA	Malonyl-CoA	
M5	Malonyl-CoA	Malonyl-CoA	Malonyl-CoA	
M6	Methylmanonyl-CoA	Methylmanonyl-CoA[a]	Methylmanonyl-CoA	Yes
M7	Malonyl-CoA	Malonyl-CoA	Malonyl-CoA	
M8-A	Alanine	Glycine/Alanine	-	
M9	Malonyl-CoA	Malonyl-CoA	Malonyl-CoA	
M10	Methylmanonyl-CoA	Methylmanonyl-CoA[a]	Methylmanonyl-CoA	Yes
M11	Methylmanonyl-CoA	Methylmanonyl-CoA[a]	Methylmanonyl-CoA	Yes
M12	Methylmanonyl-CoA	Methylmanonyl-CoA[a]	Methylmanonyl-CoA	Yes

[a] Monomer specificity prediction by antiSMASH are methylmalonyl-CoA, but according to active site details in antiSMASH these AT domains are unspecific.

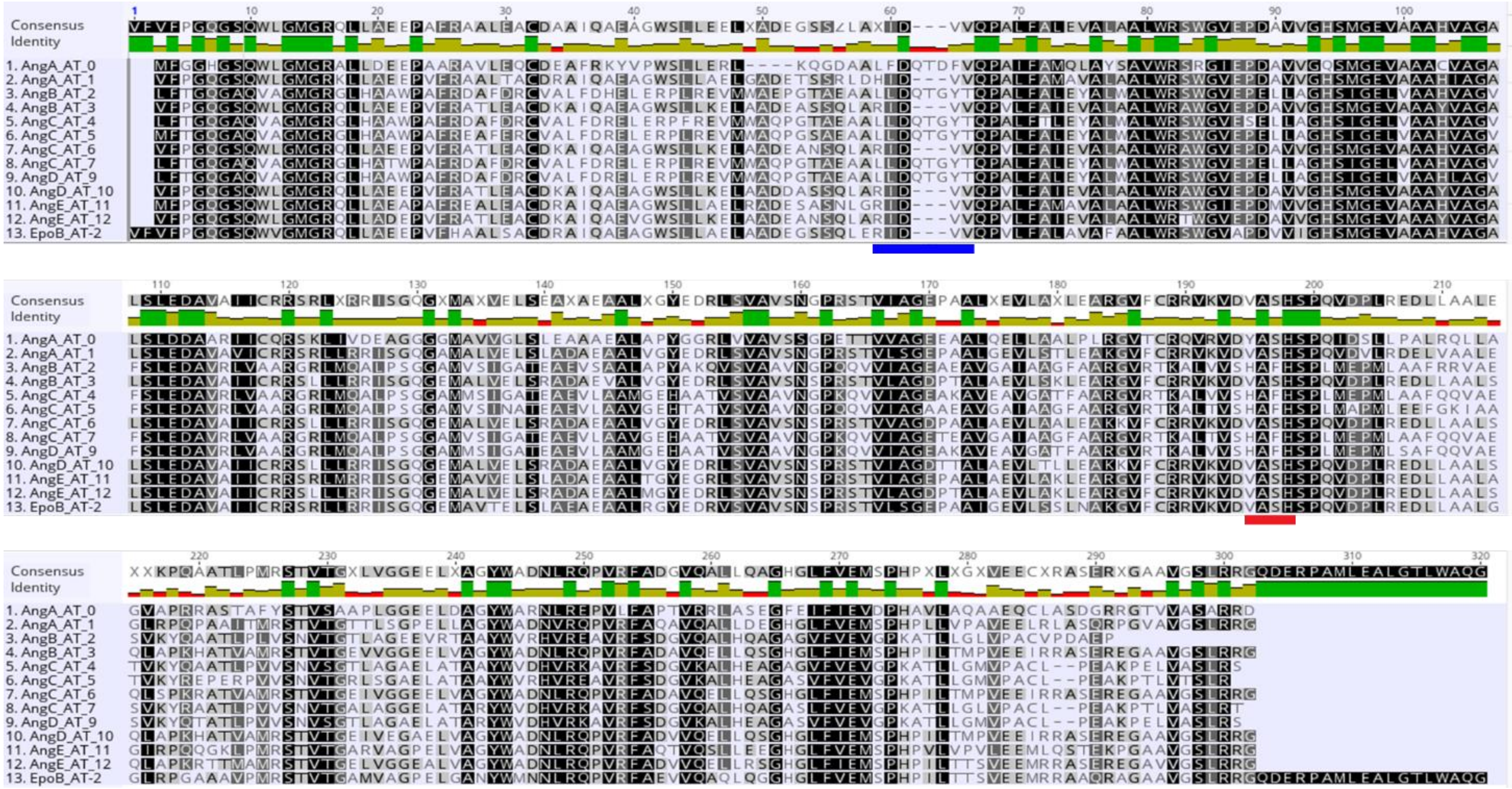

Figure S 4: Alignment of the AT domains in the *ang* BGC and the AT domain in module 2 of the *epo* BGC in *Sorangium cellulosum* So ce90. The fingerprint region for the distinction of the substrate specificity between malonyl-CoA (HAFH) and methylmalonyl-CoA (Y/VASH) is highlighted with a red bar, the putative region indicating a substrate specificity of methylmalonyl-CoA and ethylmalonyl-CoA (RiDVV) is highlighted with a blue bar.

Table S 23: Reductive loops in angiolam BGC and *in silico* predictions of the stereochemistry of angiolam A

Module	Position	Final stereochemistry	Relative stereochemistry in module	AntiSMASH[6]	KR type[9]	KR HMM[10]	DH fingerprint[7]	ER fingerprint[11]
1	C3	double bond: *E*	double bond: *E*	D (KR)	B1: *R*	D	*E* (B1 KR)	Inactive[a]
	C4	double bond: *E*	double bond: *E*	*R* (ER)	B1: *R*	L	*E* (B1 KR)	Inactive[a]
2	C5	*S*	*S*/D	D (KR)	A1: *S*	L	No DH	No ER
	C6	-	-	-	-	-	No DH	No ER
3	C7	-	-	D (KR)	B1: *R*	D	*E* (B1 KR)	-
	C8	*R*	*R*/D	*R* (ER)	B1: *R*	D	*E* (B1 KR)	*R*
4	C9	double bond: *E*	double bond: *E*	D (KR)	B1: *R*	D	*E* (B1 KR)	No ER
	C10	double bond: *E*	double bond: *E*	-	-	-	*E* (B1 KR)	No ER
5	C11	*S*	*S*/D	D (KR)	B1: *R*	D	No DH	No ER
	C12	-	-	-	-	-	No DH	No ER
6	C13	-		D (KR)	B1: *R*	D	*E* (B1 KR)	-
	C14	*R*	*R*/D	R (ER)	B1: *R*	D	*E* (B1 KR)	*R*
7	C15	-	-	-	No KR	No KR	Inactive[b]	No ER
	C16	-	-	-	No KR	No KR	Inactive[b]	No ER
8	C17	-	-	-	NRPS module	NRPS module	NRPS module	NRPS module
	C18	*S*	*S*	-				
9	C19	-	-	D (KR)	B1: *R*	D	*E* (B1 KR)	-
	C20	-	-	S (ER)	-	-	*E* (B1 KR)	S
10	C21	-		L (KR)	C, unspecified[c]	D	No DH	-
	C22	*S*	*R*/D	*R* (ER)	C, unspecified[c]	D	No DH	Unclear[d]
11	C23	*R*	*S*/L	L (KR)	A1: *S*	L	No DH	No ER
	C24	*S*	*R*/D	-	A1: *R*	D	No DH	No ER
12	C25	double bond: *E*	double bond: *E*	D (KR)	B1: *R*	D	*E* (B1 KR)	Inactive[a]
	C26	double bond: *E*	double bond: *E*	inconclusive (ER)	B1: *R*	D	*E* (B1 KR)	Inactive[a]

In-silico predictions of modules 1-12 and the positions the respective KR, DH and ER domains effect. Domains that determine the stereochemistry of the respective positions are marked in green, when they are fitting. In modules 2 and 5 the stereochemistry predictions of the KR domains differed between prediction tools and in 10 only the predicted stereochemistries of antiSMASH were correct. This might be explained by the limited number of domains that were available when the rules for the manually applied classification systems for KR[9,10] and ER[11] domains were formulated.

[a] ER domains in modules 1 and 12 are lacking the NADPH binding site needed for their reductive activities.
[b] The DH domain in module 7 does not possess the catalytic triad H, P, G.
[c] The KR domain in module 10 does not belong to type A or type B KRs, as it possesses neither a W residue in the catalytic region nor a LDD motif in the loop. As the catalytic region features an intact YAAAN motif, it belongs neither to the C1 nor the C2 type KRs[9].
[d] The ER domain in module 10 does not possess the typical fingerprins leading to neither a (2*S*)- nor a (2*R*)-configuration.

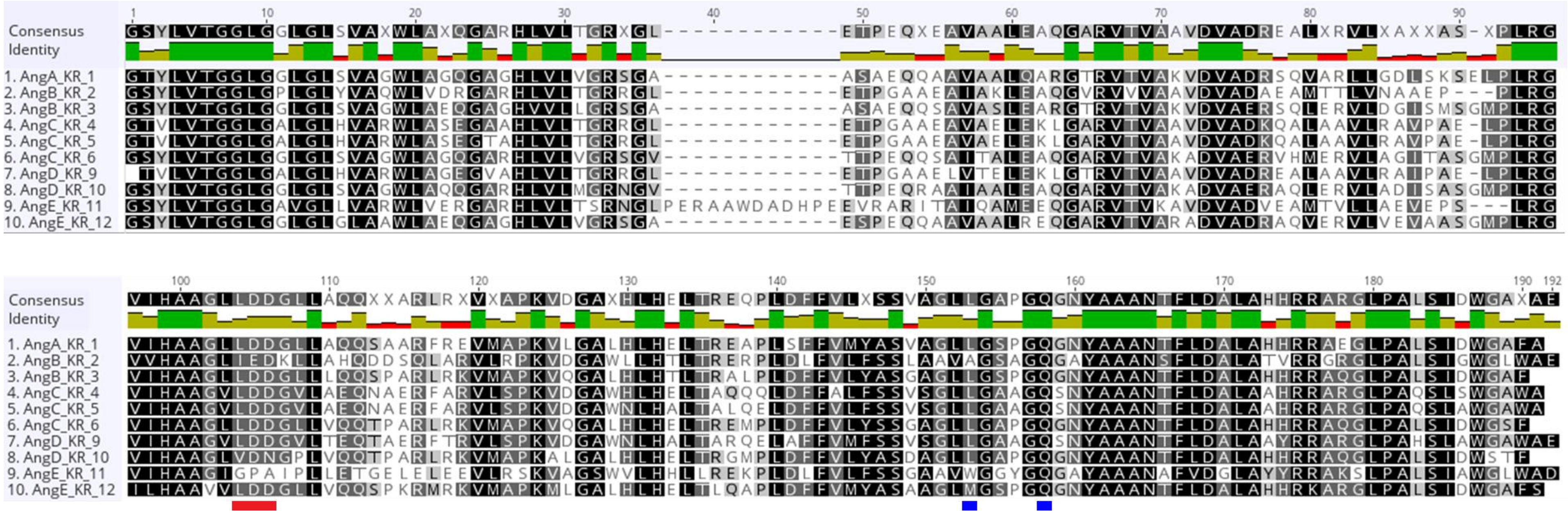

Figure S 5: Alignment of the KR domains in the *ang* BGC. The fingerprint region indicative for B-type KR domains (LDD) is highlighted with a red bar, regions that distinguish A1 and A2 type KR domains are highlighted with a blue bar.

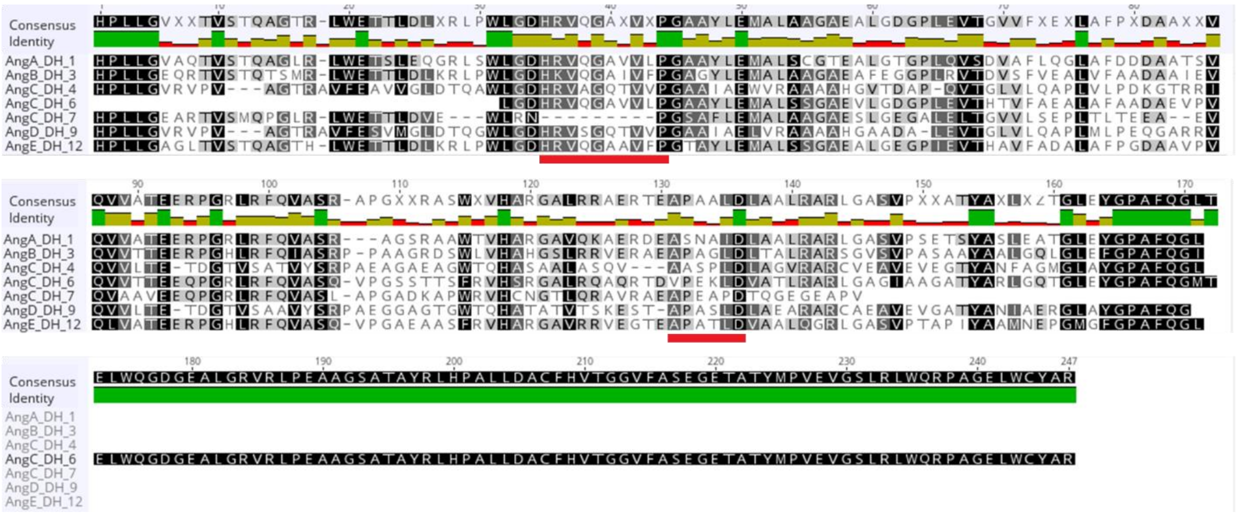

Figure S 6: Alignment of the DH domains in the *ang* BGC. The catalytic regions (HxxxGxxxxP and HPALL**D**) needed for the elimination of water, are highlighted with red bars.

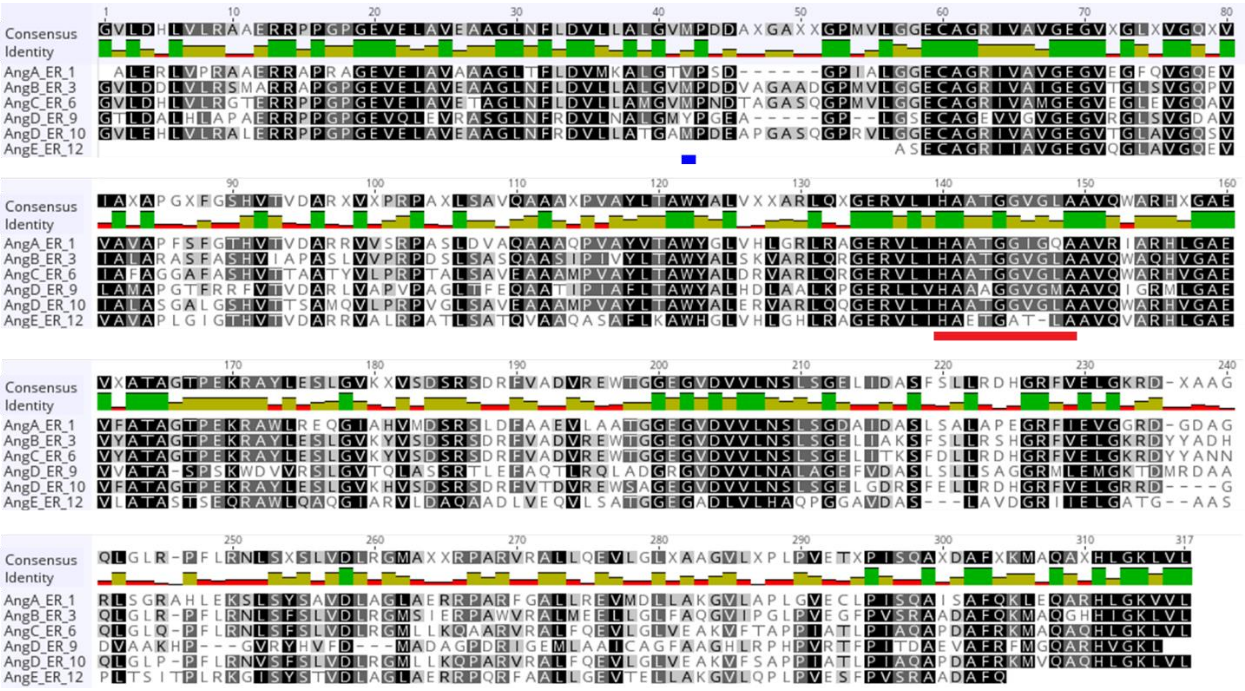

Figure S 7: Alignment of the ER domains in the *ang* BGC. The NADPH binding site (HaAtGGVGxA) is highlighted with a red bar. The tyrosine residue, catalysing the *S*-conformation of residues bound to position two in a given module is indicated with a blue bar.

2.3.1 Origin of ethyl-residues in angiolams D and F

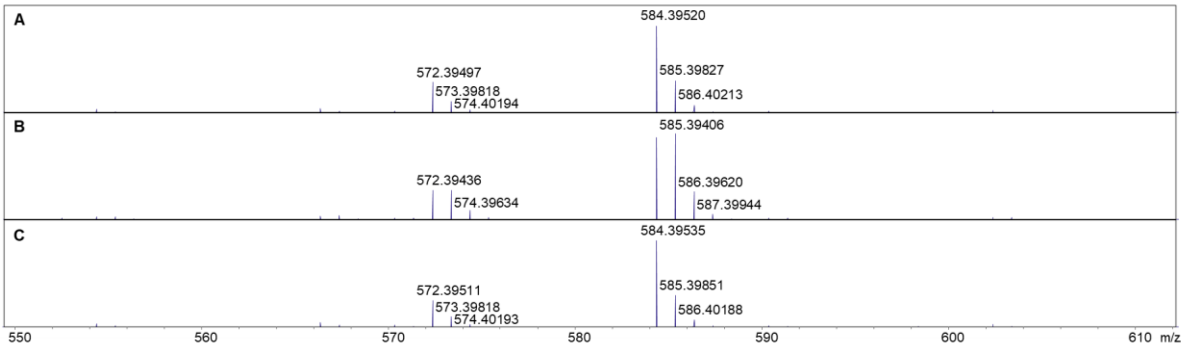

Figure S 8: Mass spectra of angiolam D from cultivations with labelled precursors. Isotope patterns of angiolams C (*m/z* 572.39) and D (*m/z* 584.39) cultivations of MCy12733 in Cy/H medium as control (A), with L-alanine ^{15}N (B) and L-methionine-(methyl ^{13}C) (C).

Table S 24: Production of angiolams A, D and F in Cy/H medium and with additional acetate, propionate, butyrate or L-valine

	Cy/H		Cy/H-Acetate[a]		Cy/H-Propionate[b]		Cy/H-Butyrate[c]		Cy/H-Valine[d]	
	Area	Average area	Area	Average area	Area	Average area	Area	Average area	Area	Average area
	25655576		19816828		11008598		9136835		7678847	
**A**	25483662	25355307	15746595	17255239	4027166	8350200	7346901	8042633	9128582	7753207
	24926684		16202293		10014836		7644162		6452191	
	572186		357620		58126		892692		3241019	
**D**	598411	569492	286440	316774	332349	148363	734559	794822	3977963	3365068
	537879		306263		54615		757215		2876223	
	0		0		0		82104		871836	
**F**	0	0	0	0	0	0	66037	71727	1074687	927082
	0		0		0		67040		834723	

[a] Addition of 20 mM Na acetate to Cy/H medium.
[b] Addition of 20 mM Na propionate to Cy/H medium.
[c] Addition of 20 mM Na butyrate to Cy/H medium.
[d] Addition of 2.5 g/L L-valine to Cy/H medium.

2.4 Disruption of angiolam BGC by single cross-over inactivation

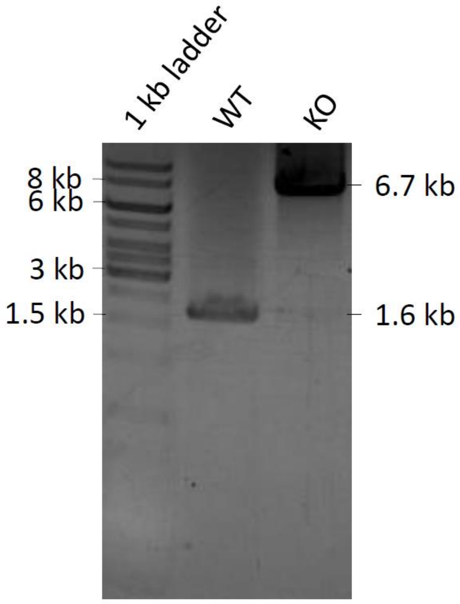

Figure S 9: Verification PCRs of integration of pTOPO-AngKO in the genome of *P. fallax* An d48. Verification PCRs for integration of pTOPO-AngKO into the genome of *P. fallax* An d48 were done with primers 48_Test_Fw and 48_Test_Rv, that bind ~ 300 bp up- and downstream the homologous region in *angB*. As shown in Figure S 4, the amplified region in the wild type strain covers 1.6 kb, while the integration mutant shows an amplified fragment of the size of ~ 6.7 kb that additionally accounts for pTOPO-AngKO with a size of 5067 bp.

3 NMR spectra employed in angiolam structure elucidation

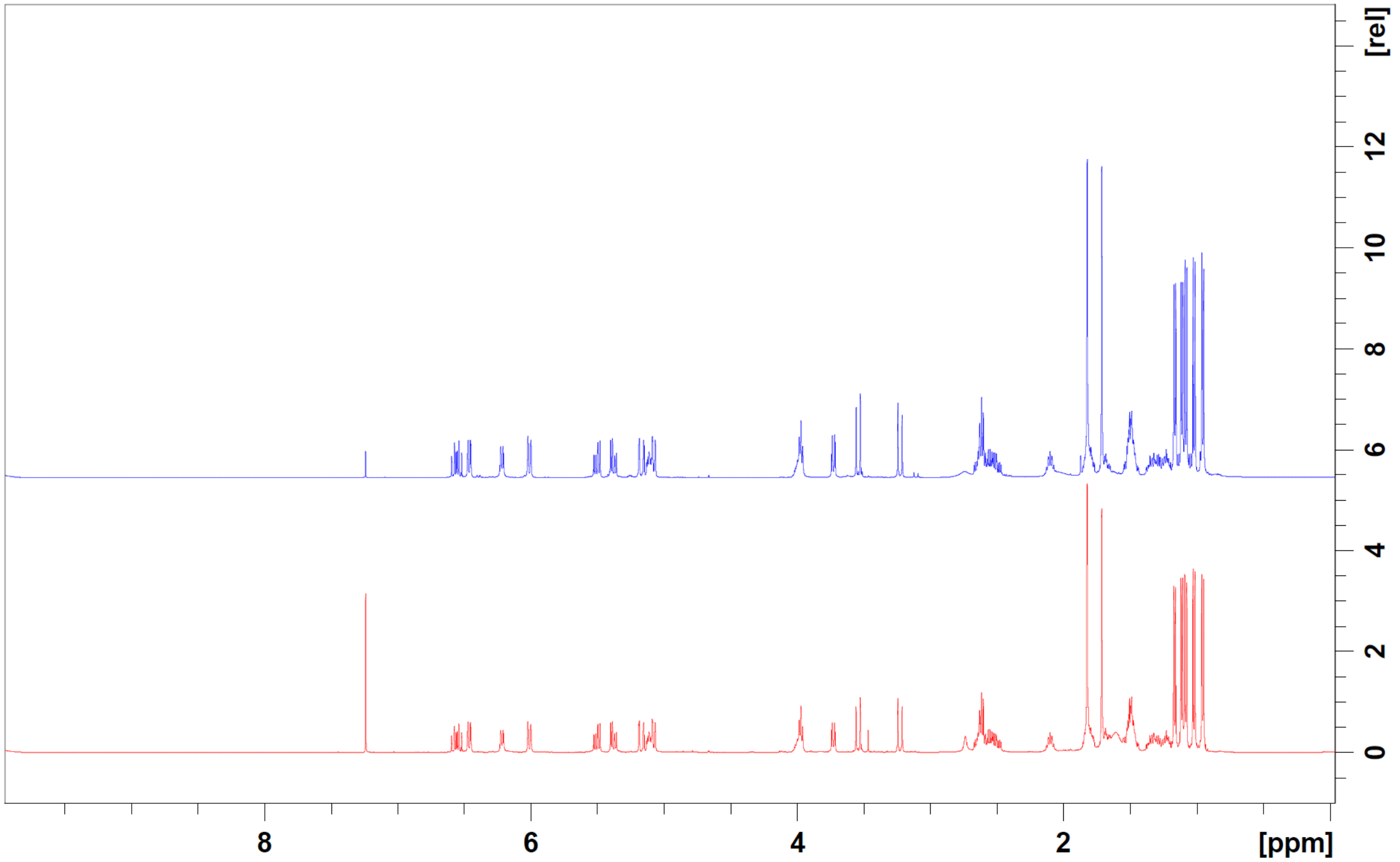

Figure S 10: Comparison of ^{1}H NMR spectra of purified angiolam A (blue) and authentic angiolam A (red) in chloroform-*d* at 500 MHz.

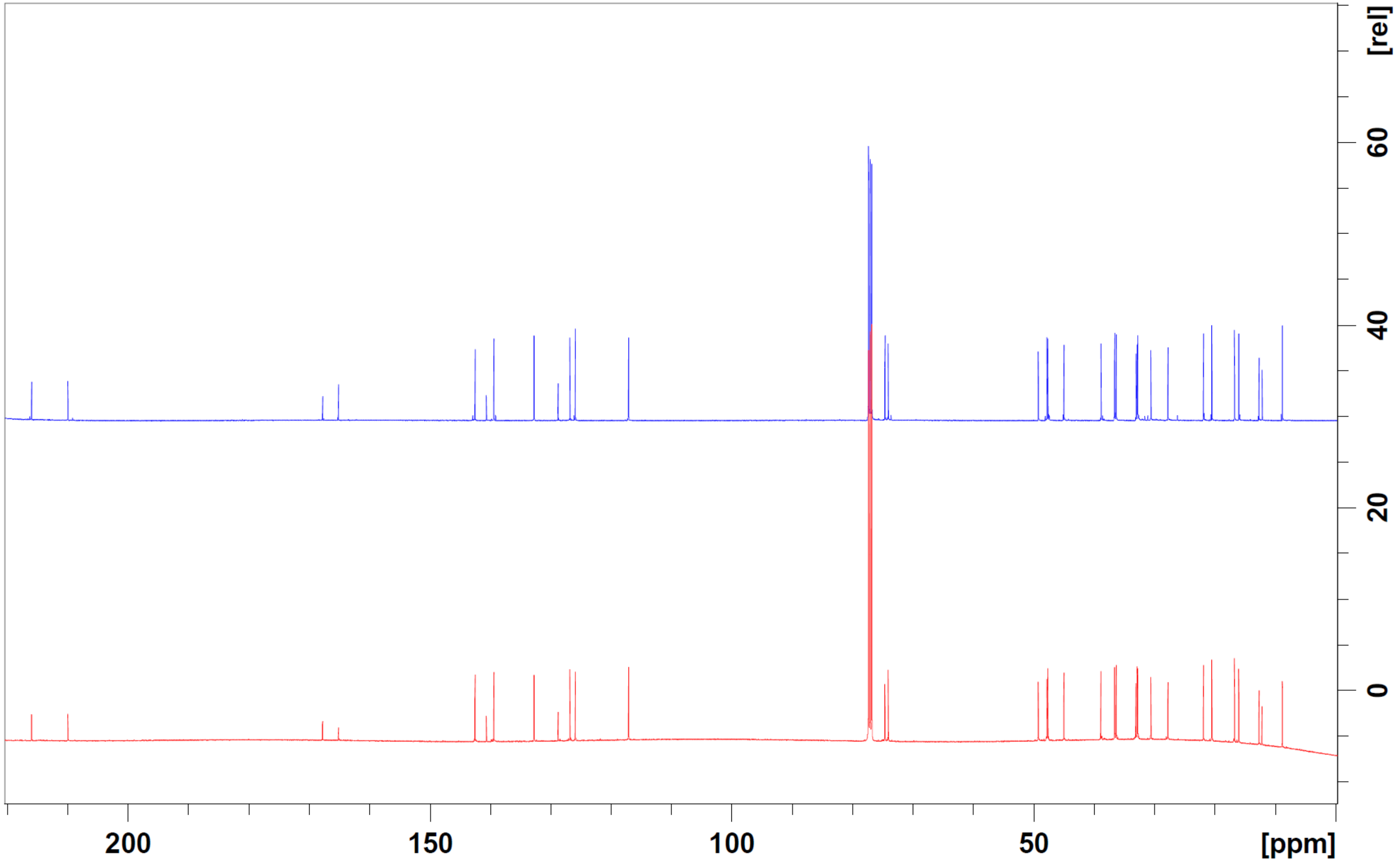

Figure S 11: Comparison of ^{13}C NMR spectra of purified angiolam A (blue) and authentic angiolam A (red) in chloroform-*d* at 125 MHz.

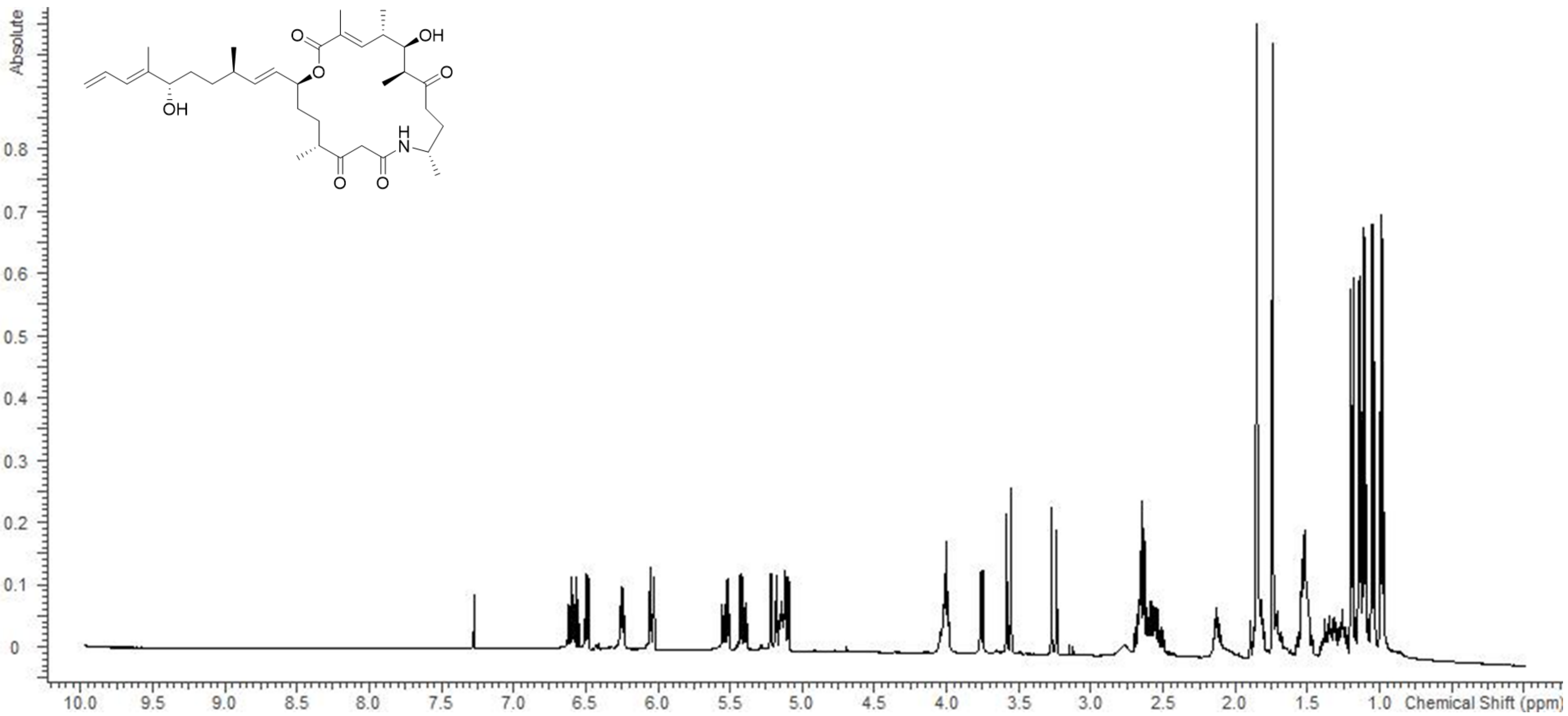

Figure S 12: ^{1}H spectrum of angiolam A (1) in chloroform-*d* at 500 MHz.

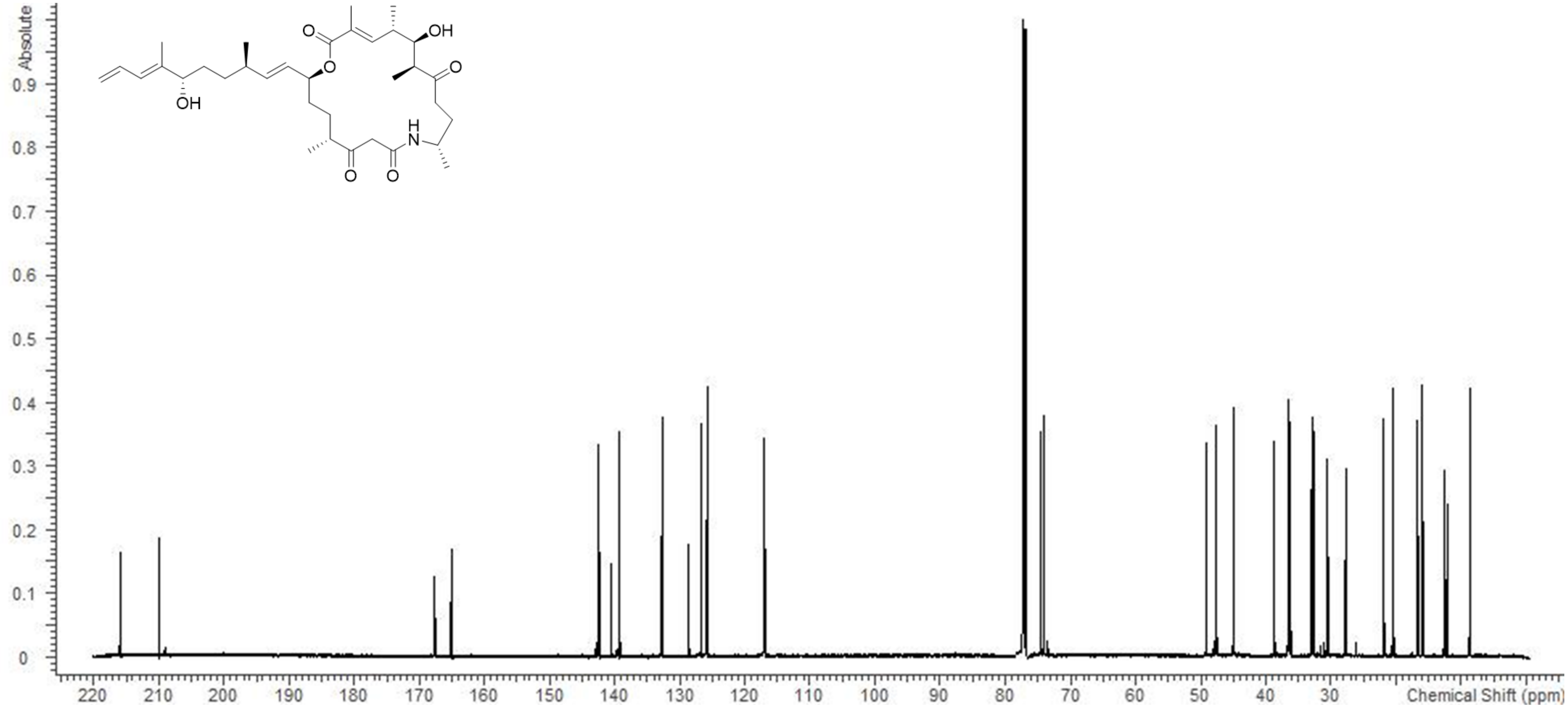

Figure S 13: ^{13}C spectrum of angiolam A (1) in chloroform-*d* at 125 MHz.

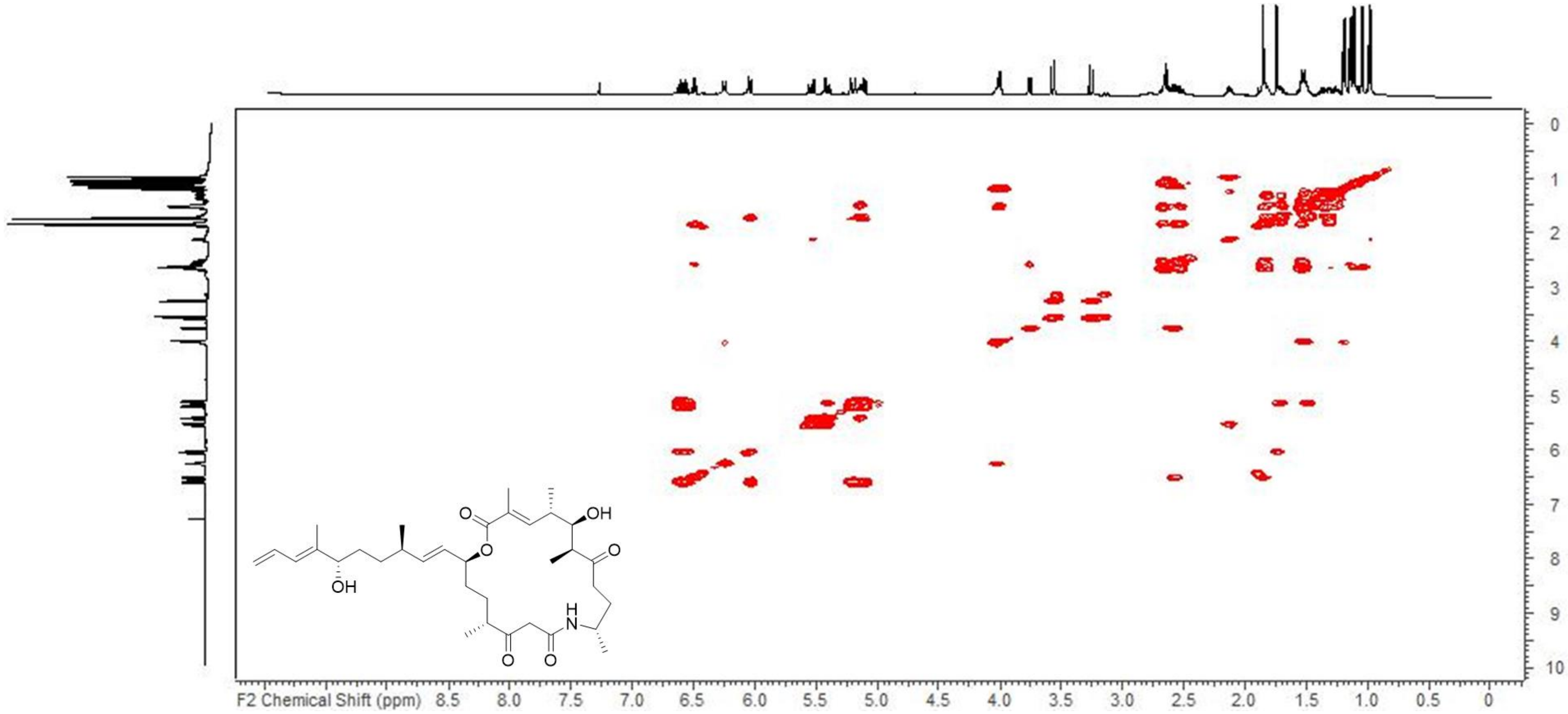

Figure S 14: COSY spectrum of angiolam A (1) in chloroform-*d* at 500 MHz.

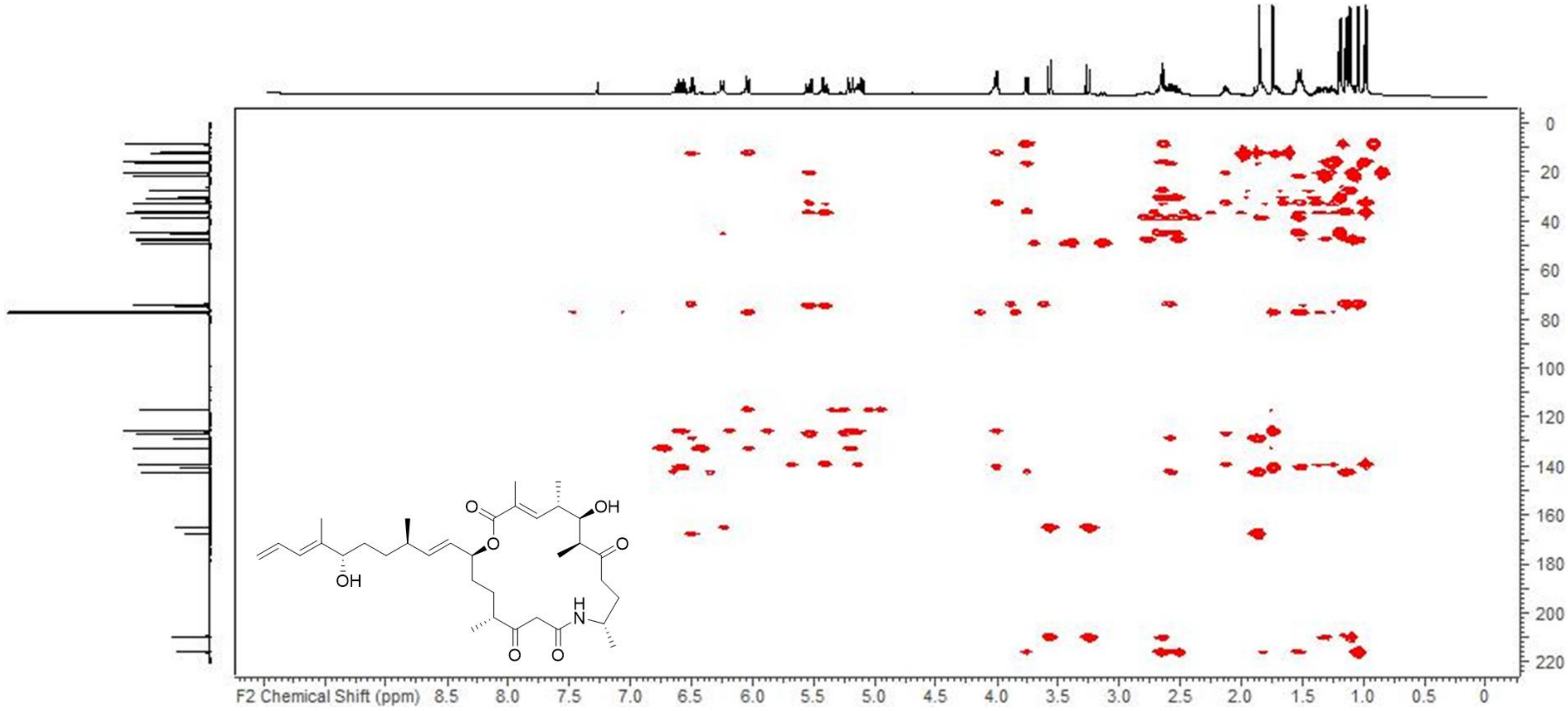

Figure S 15: HMBC spectrum of angiolam A (1) in chloroform-*d* at 125/500 (F1/F2) MHz.

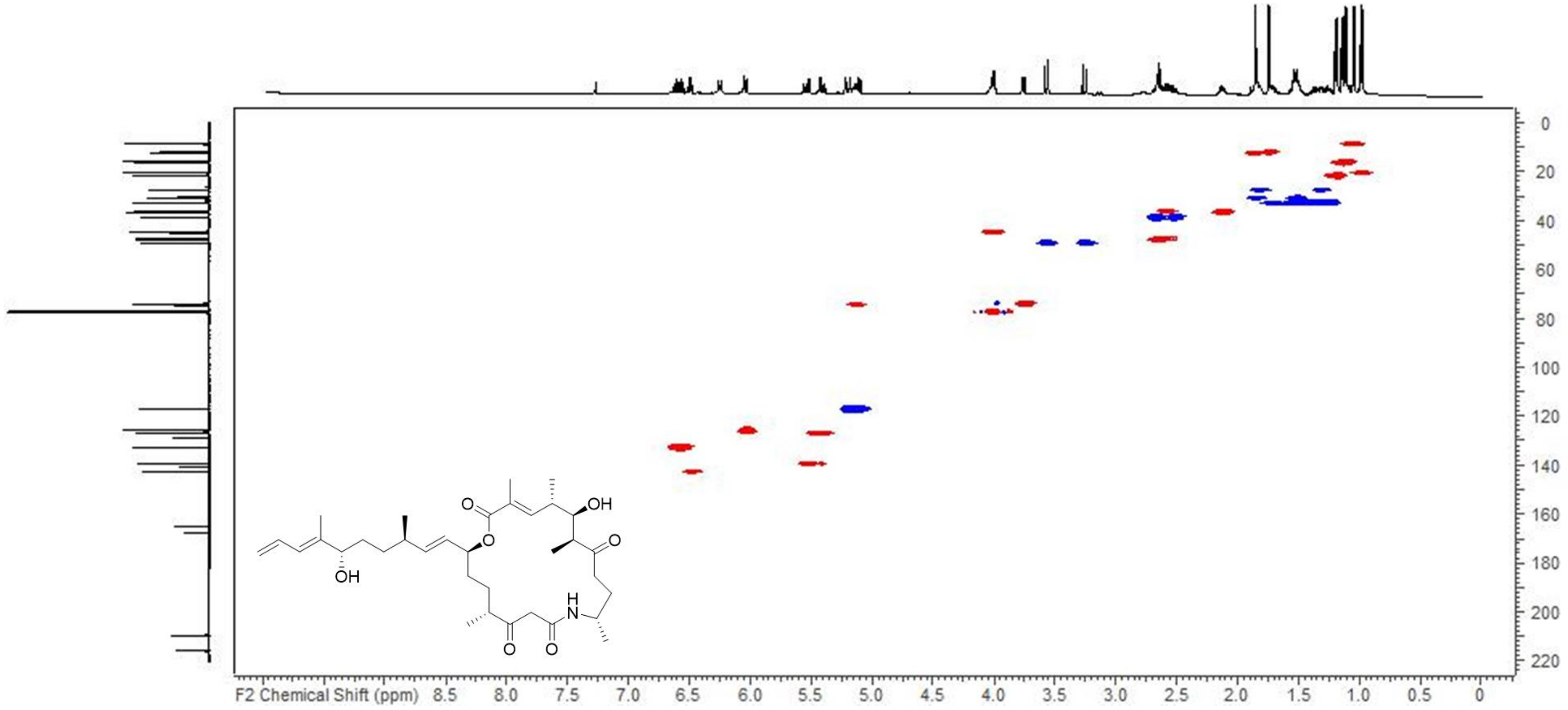

Figure S 16: HSQC spectrum of angiolam A (1) in chloroform-*d* at 125/500 (F1/F2) MHz.

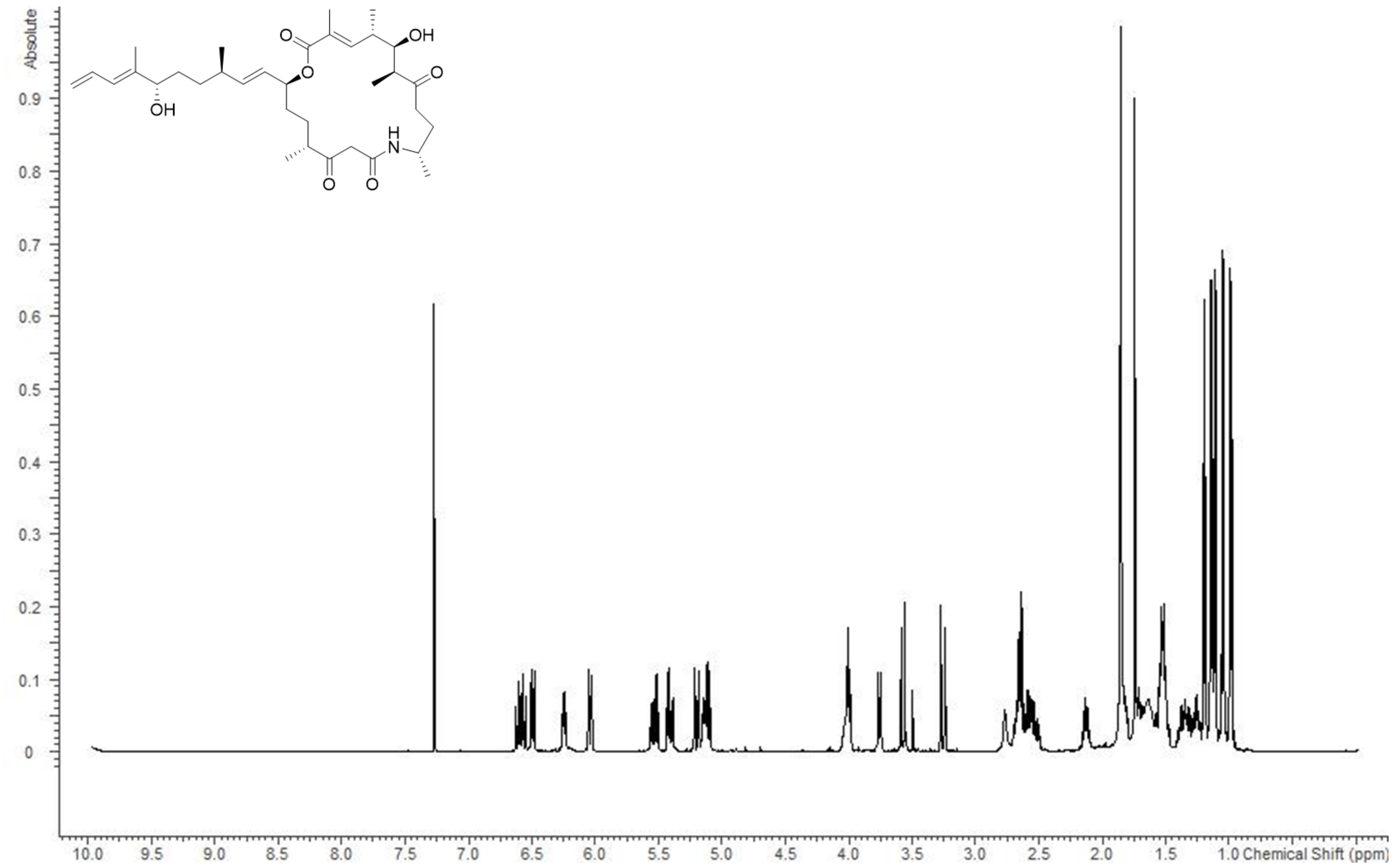

Figure S 17: ^{1}H spectrum of authentic angiolam A in chloroform-*d* at 500 MHz.

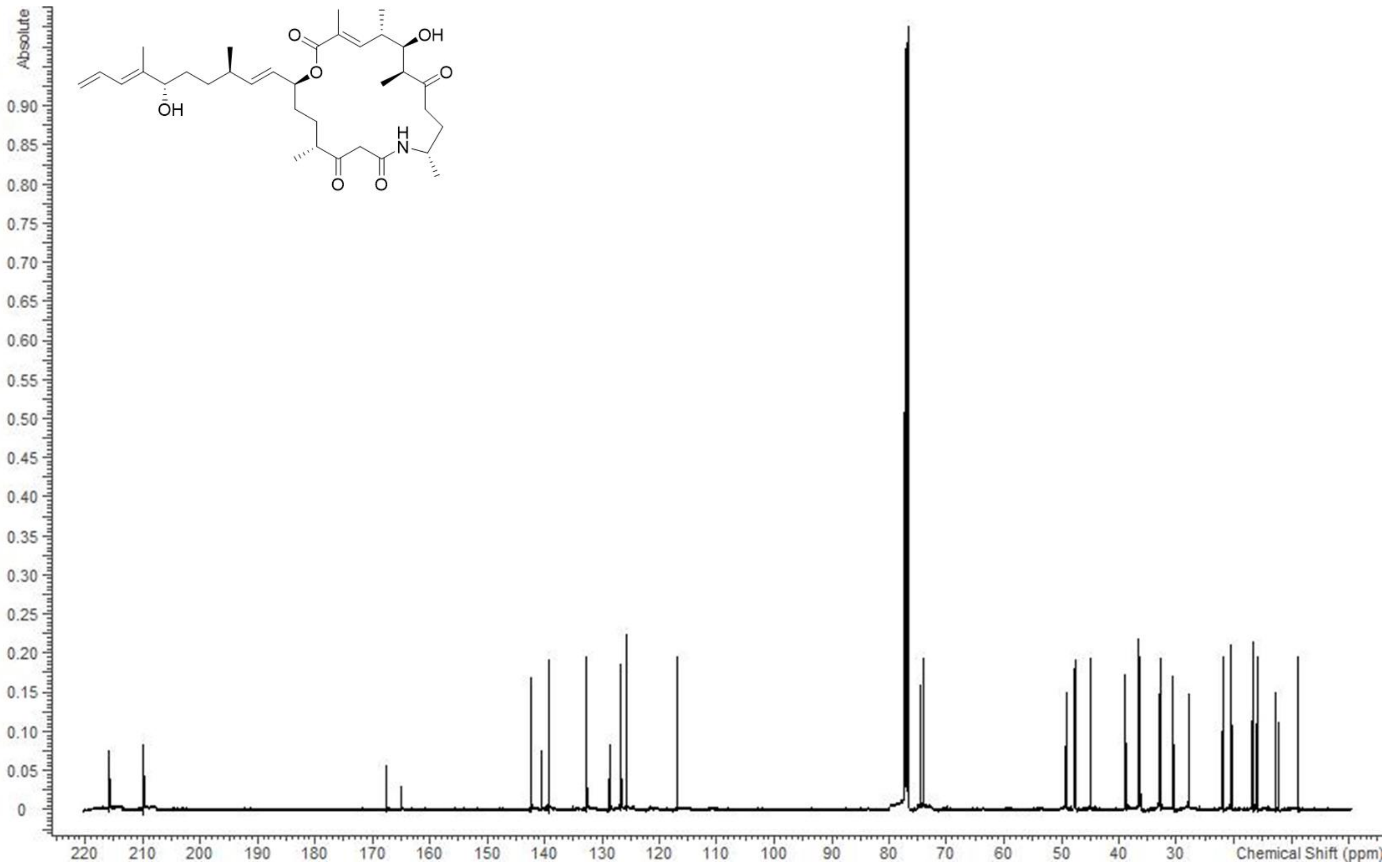

Figure S 18: ^{13}C spectrum of authentic angiolam A in chloroform-*d* at 125 MHz.

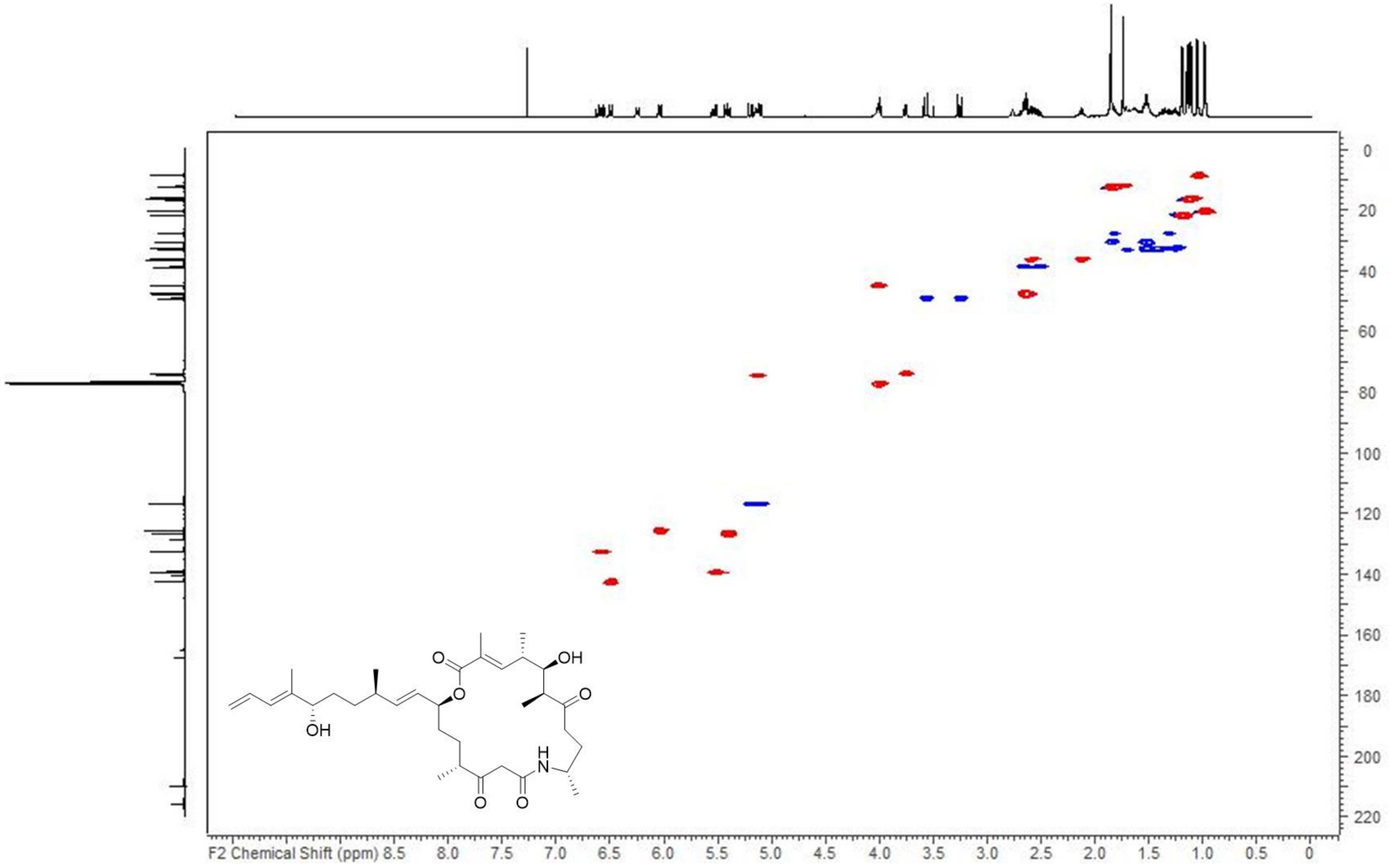

Figure S 19: HSQC spectrum of authentic angiolam A in chloroform-*d* at 125/500 (F1/F2) MHz.

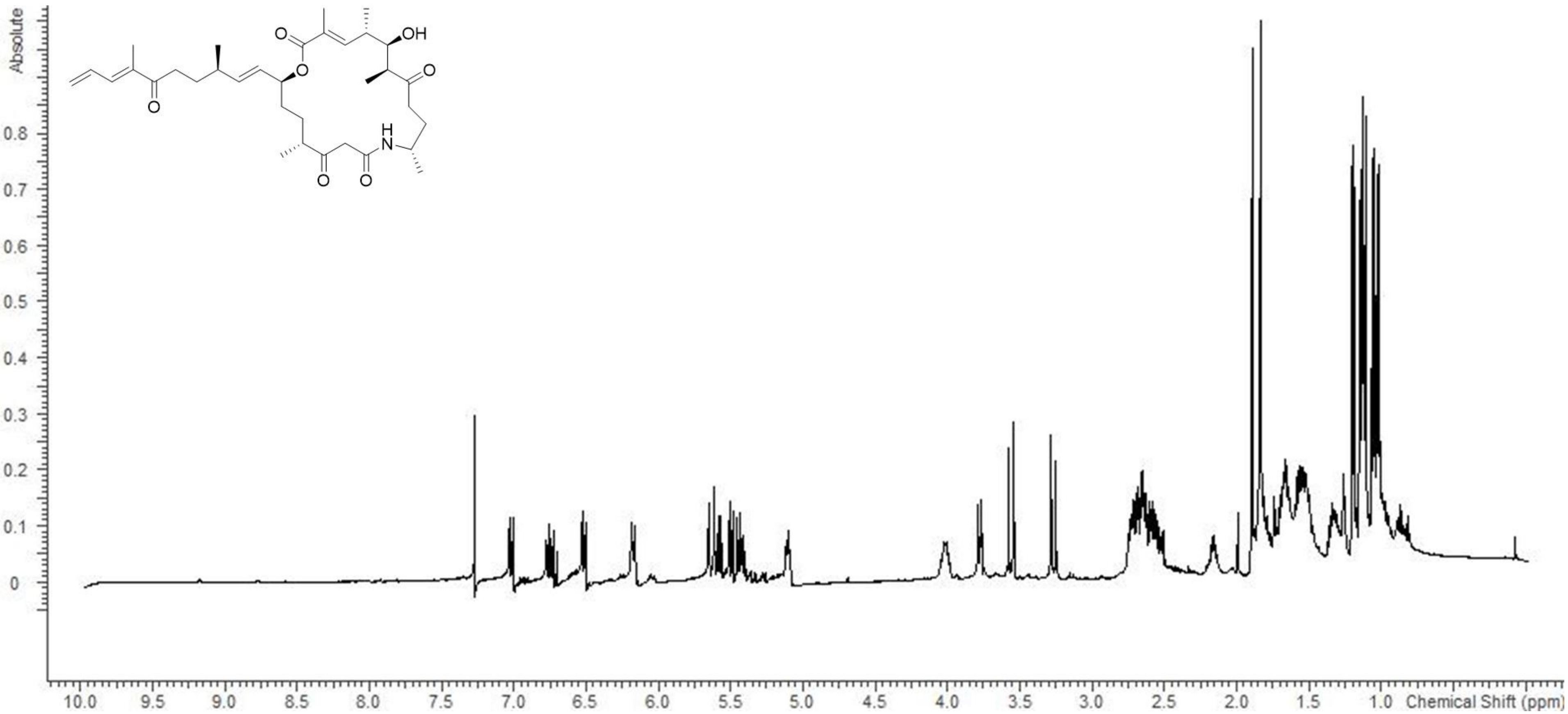

Figure S 20: ^{1}H spectrum of angiolam B (2) in chloroform-*d* at 500 MHz.

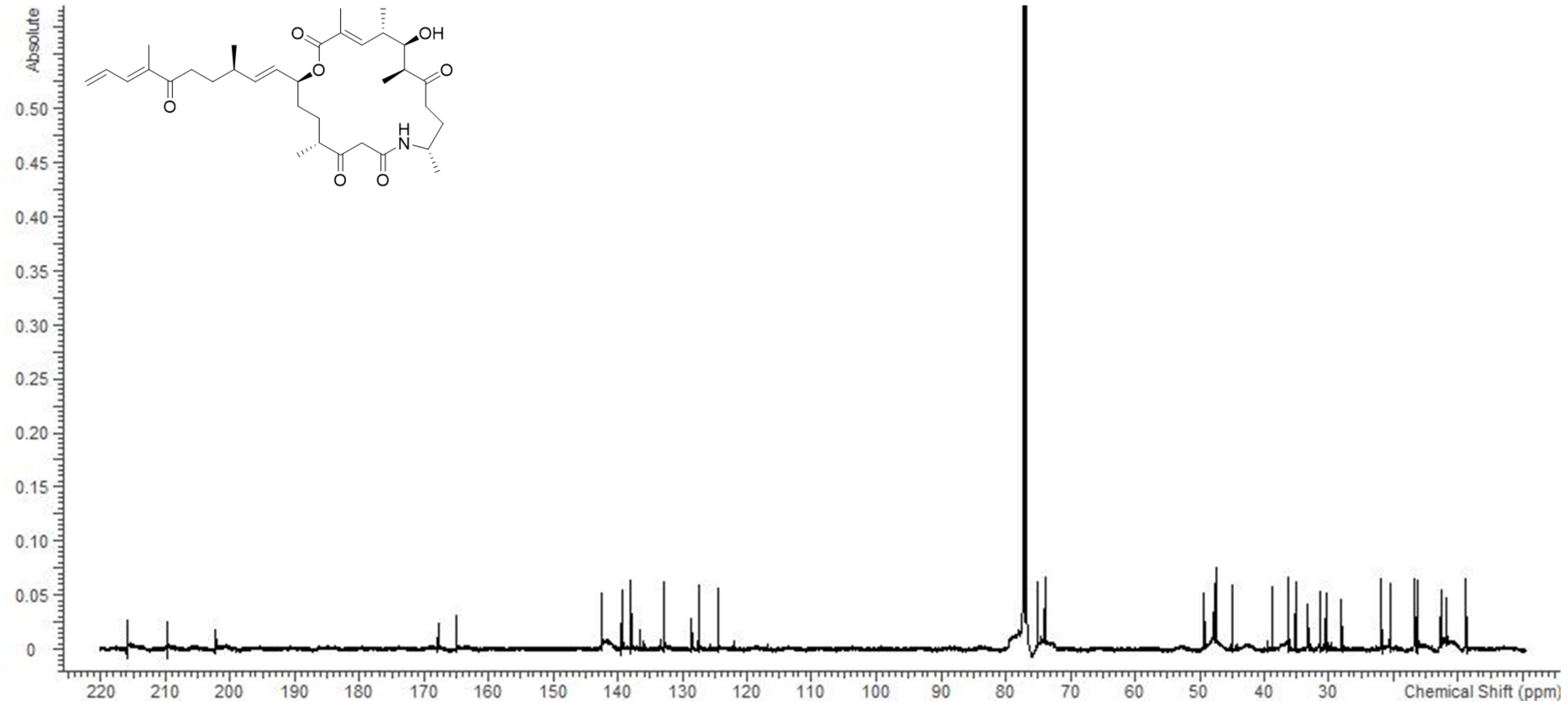

Figure S 21: ^{13}C spectrum of angiolam B (2) in chloroform-*d* at 125 MHz.

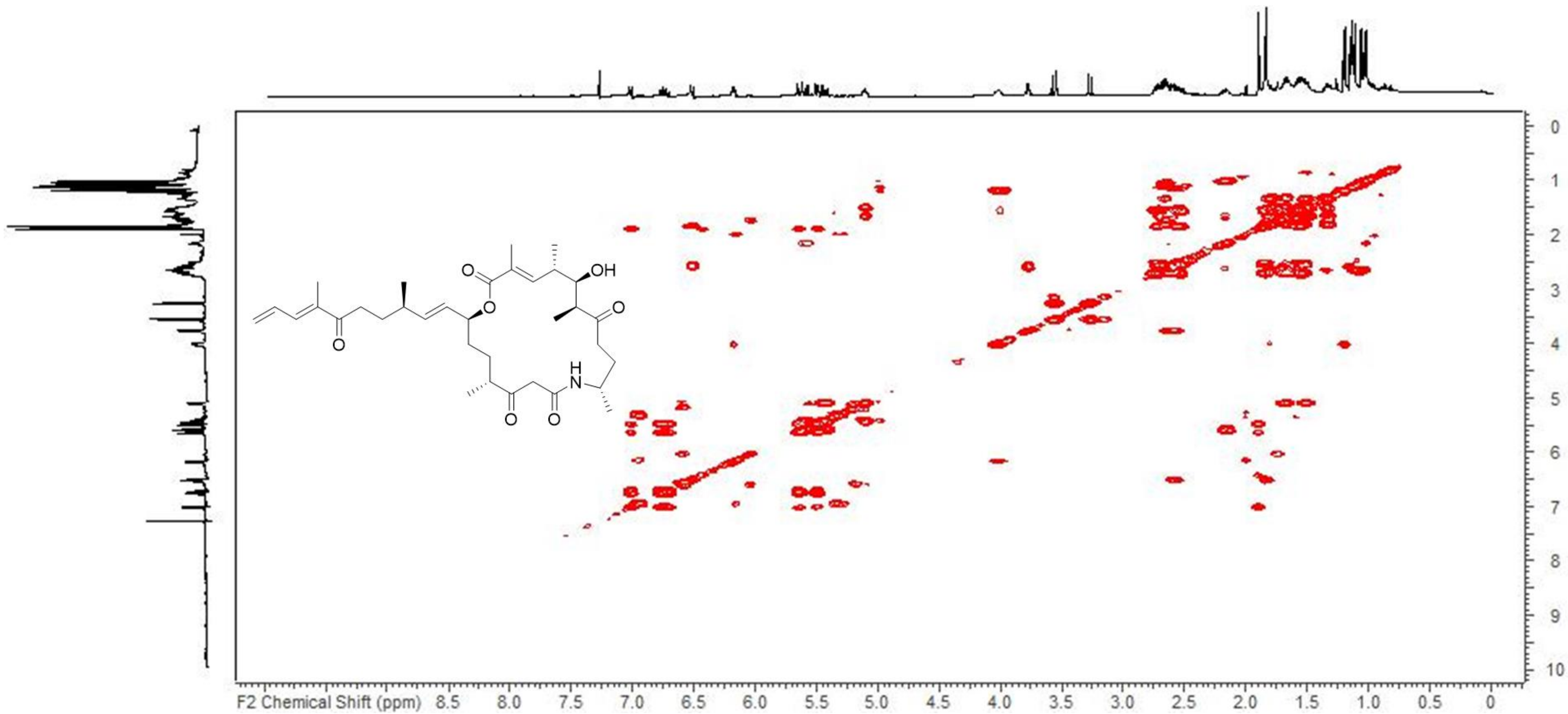

Figure S 22: COSY spectrum of angiolam B (2) in chloroform-*d* at 500 MHz.

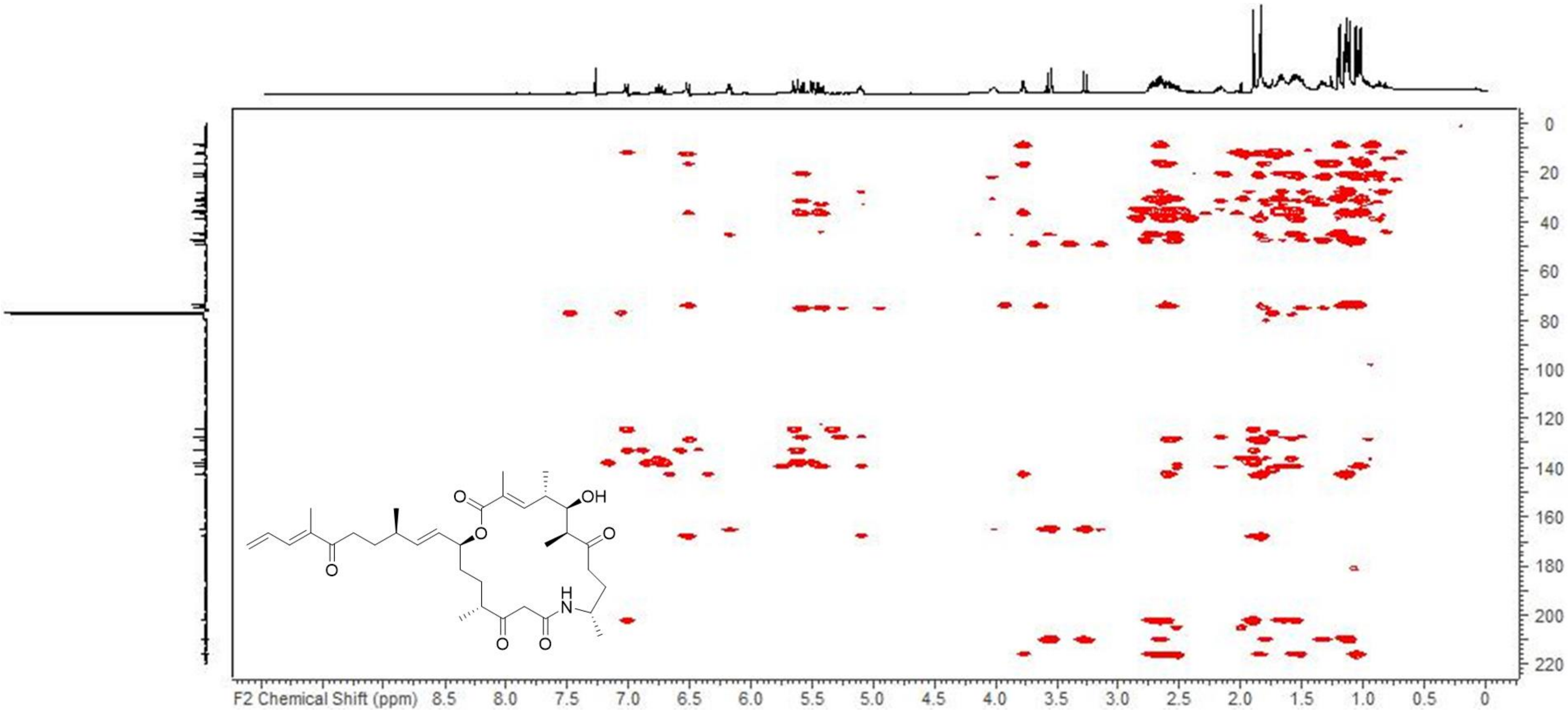

Figure S 23: HMBC spectrum of angiolam B (2) in chloroform-*d* at 125/500 (F1/F2) MHz.

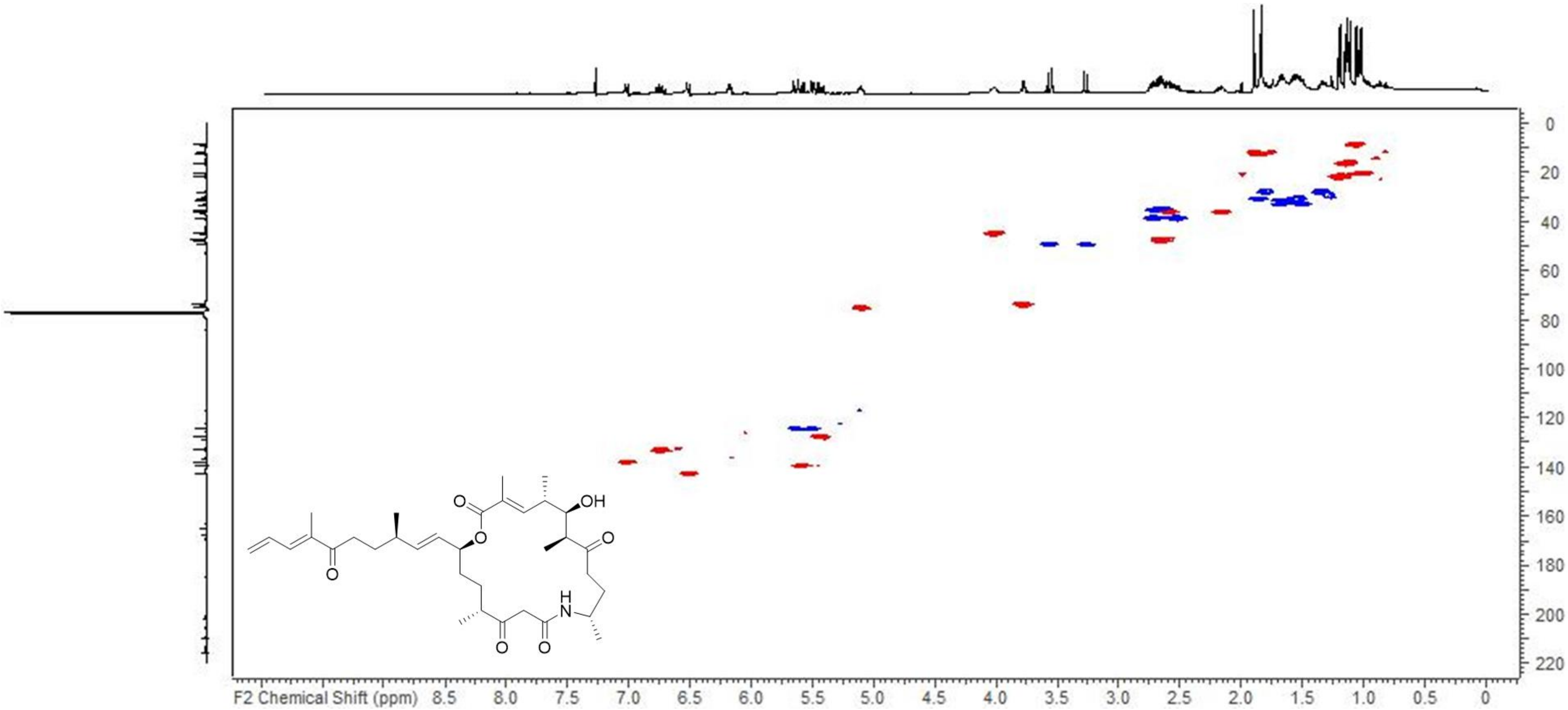

Figure S 24: HSQC spectrum of angiolam B (2) in chloroform-*d* at 125/500 (F1/F2) MHz.

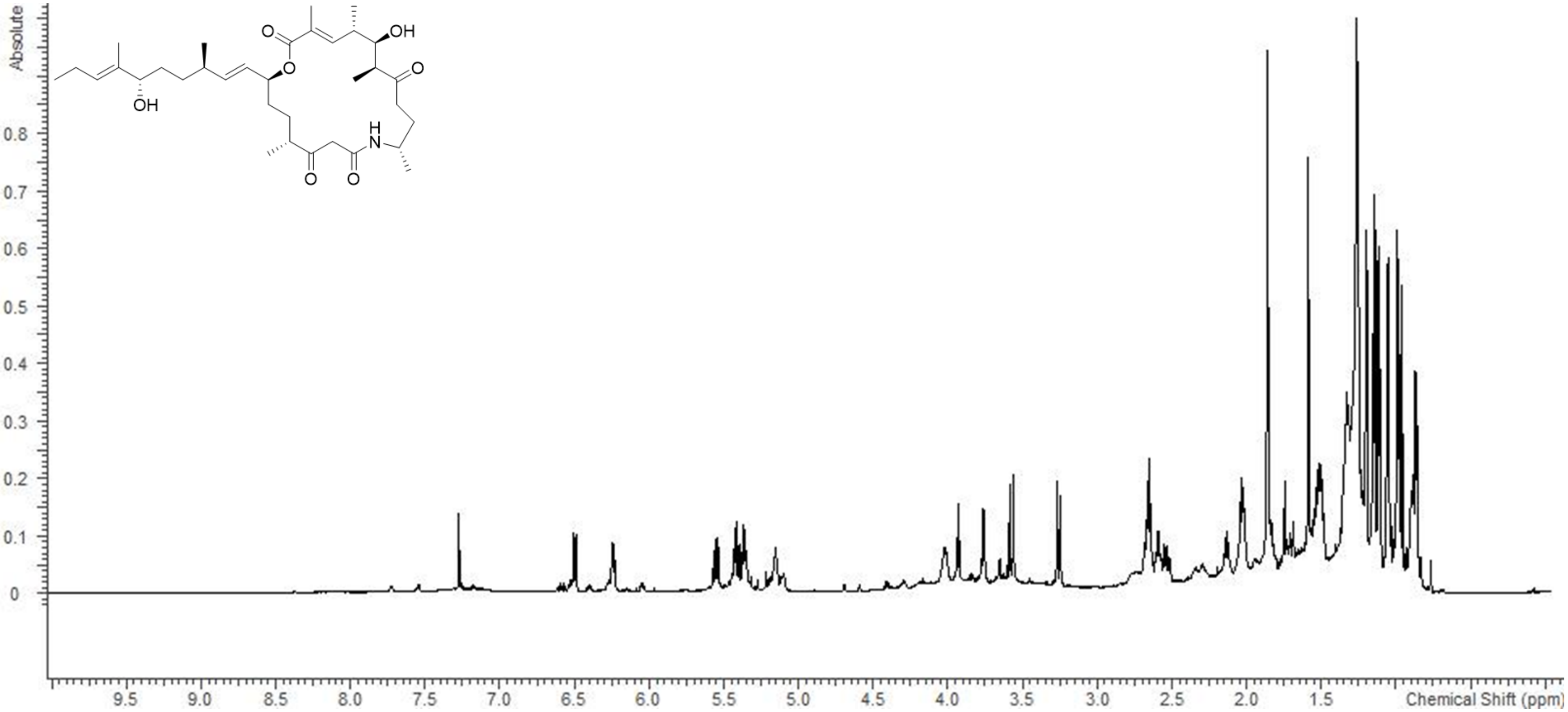

Figure S 25: ^{1}H spectrum of angiolam C (3) in chloroform-*d* at 500 MHz.

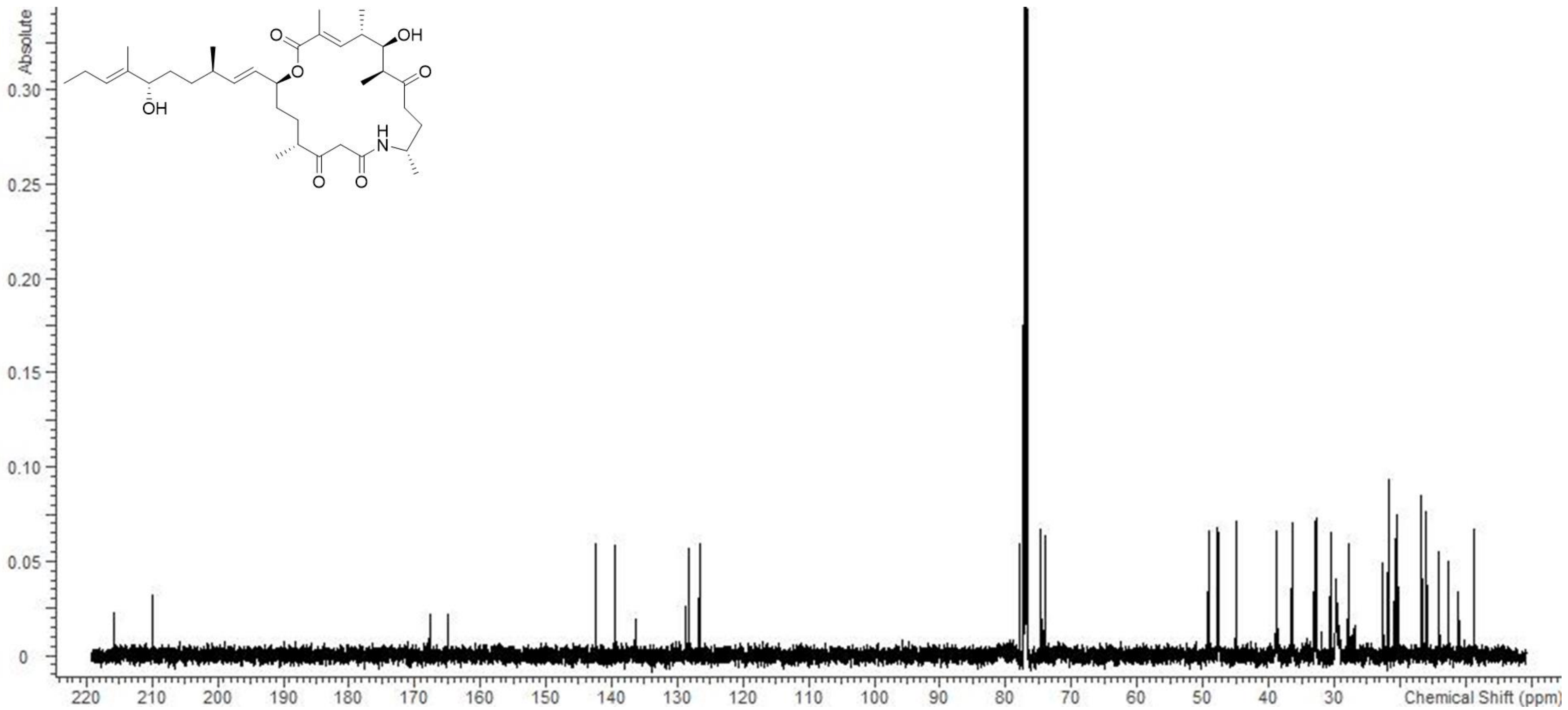

Figure S 26: ^{13}C spectrum of angiolam C (3) in chloroform-*d* at 125 MHz.

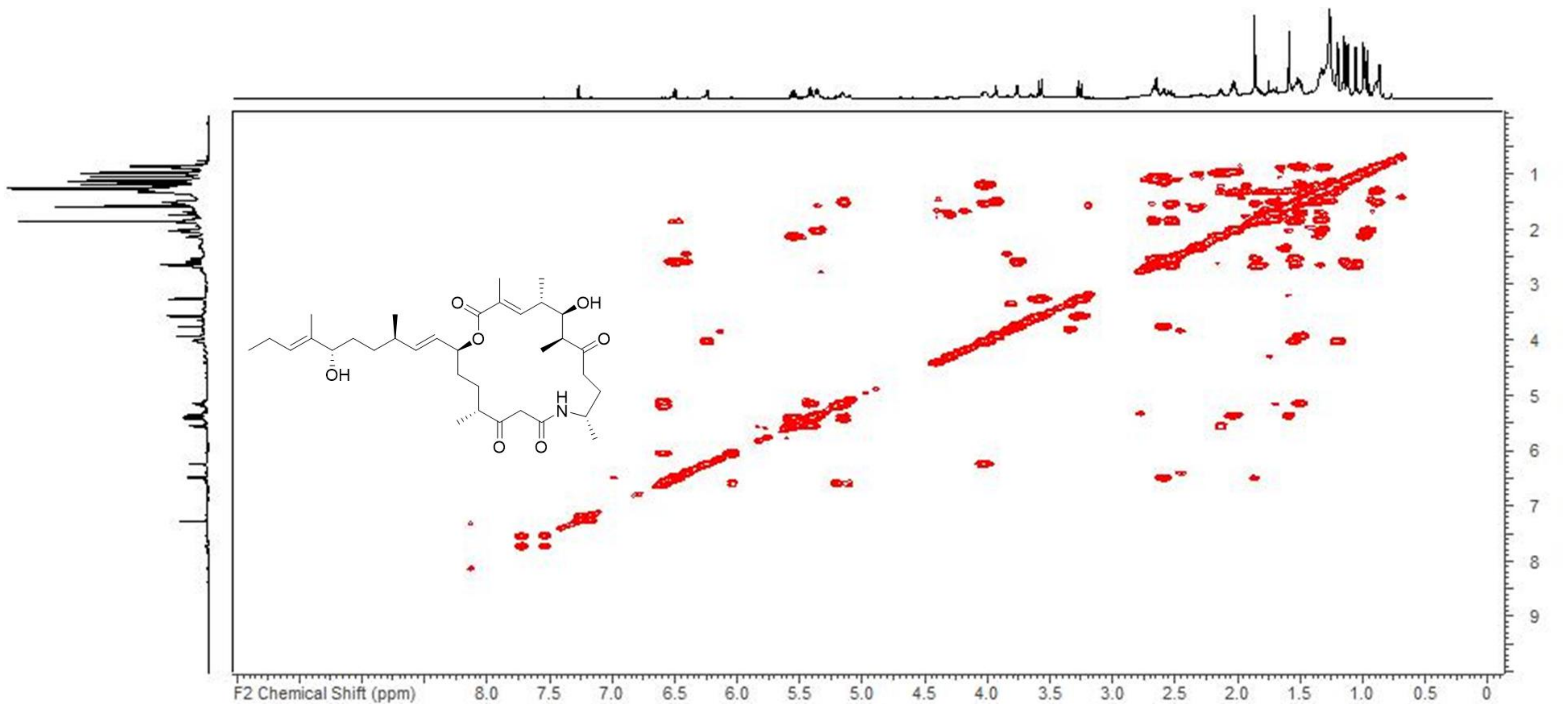

Figure S 27: COSY spectrum of angiolam C (3) in chloroform-*d* at 500 MHz.

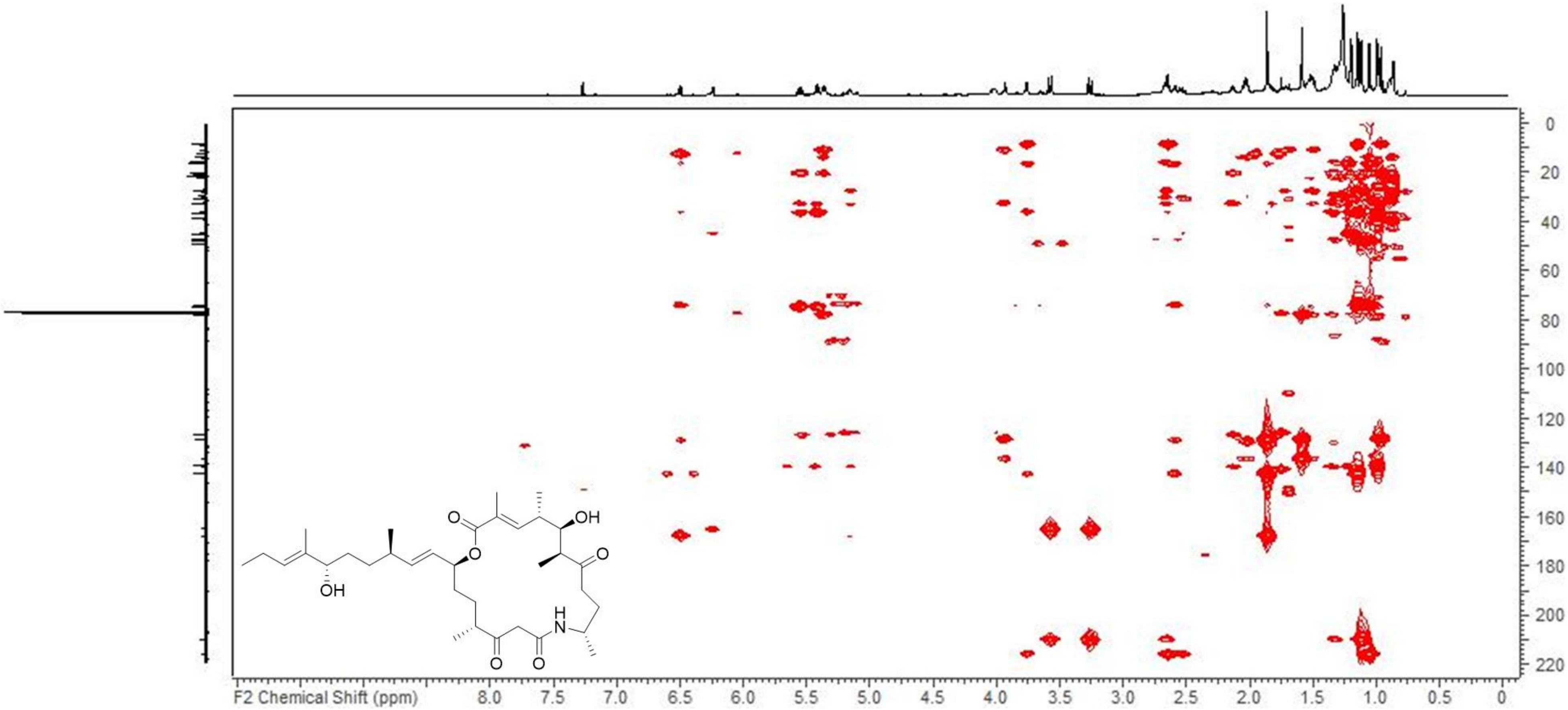

Figure S 28: HMBC spectrum of angiolam C (3) in chloroform-*d* at 125/500 (F1/F2) MHz.

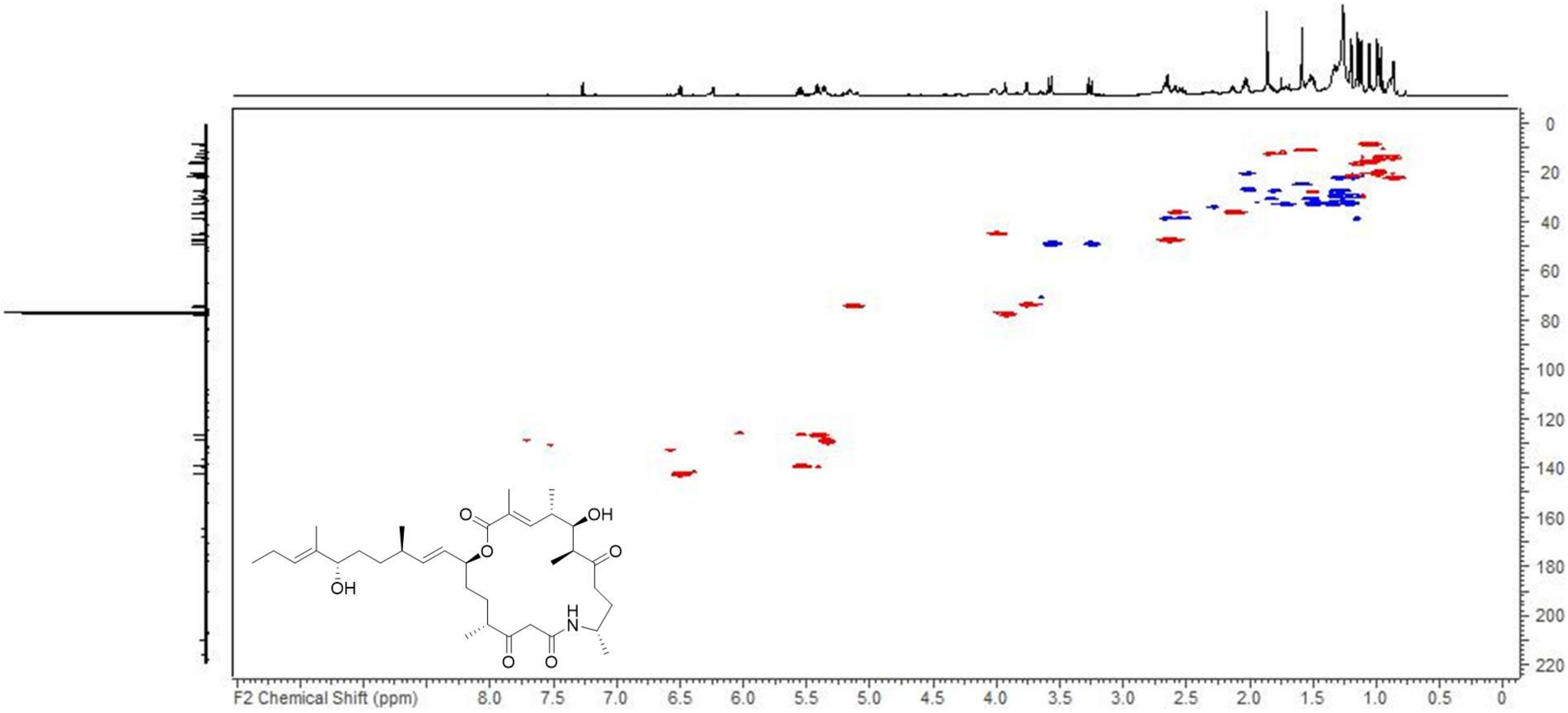

Figure S 29: HSQC spectrum of angiolam C (3) in chloroform-*d* at 125/500 (F1/F2) MHz.

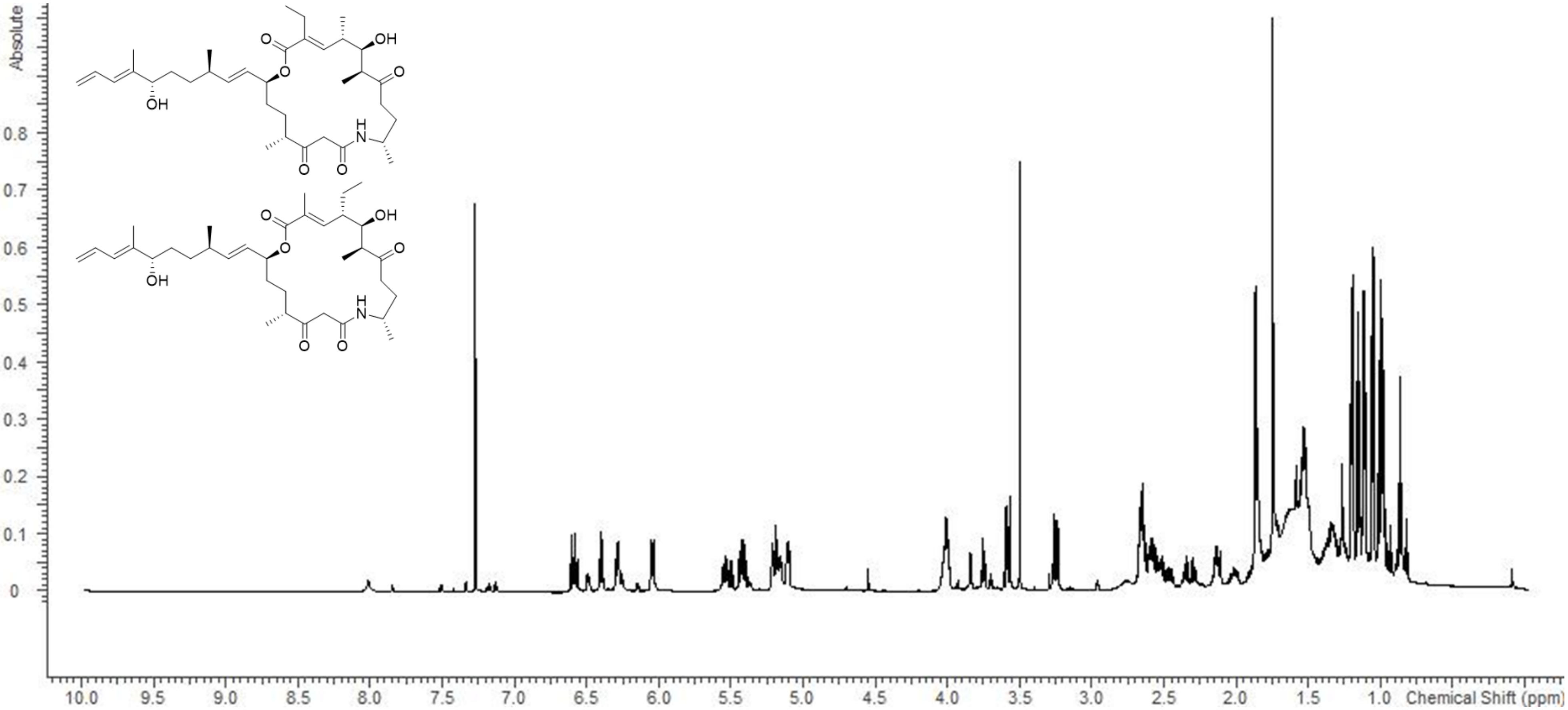

Figure S 30: ^{1}H spectrum of angiolam D_1 (4a) and D_2 (4b) in chloroform-*d* at 700 MHz.

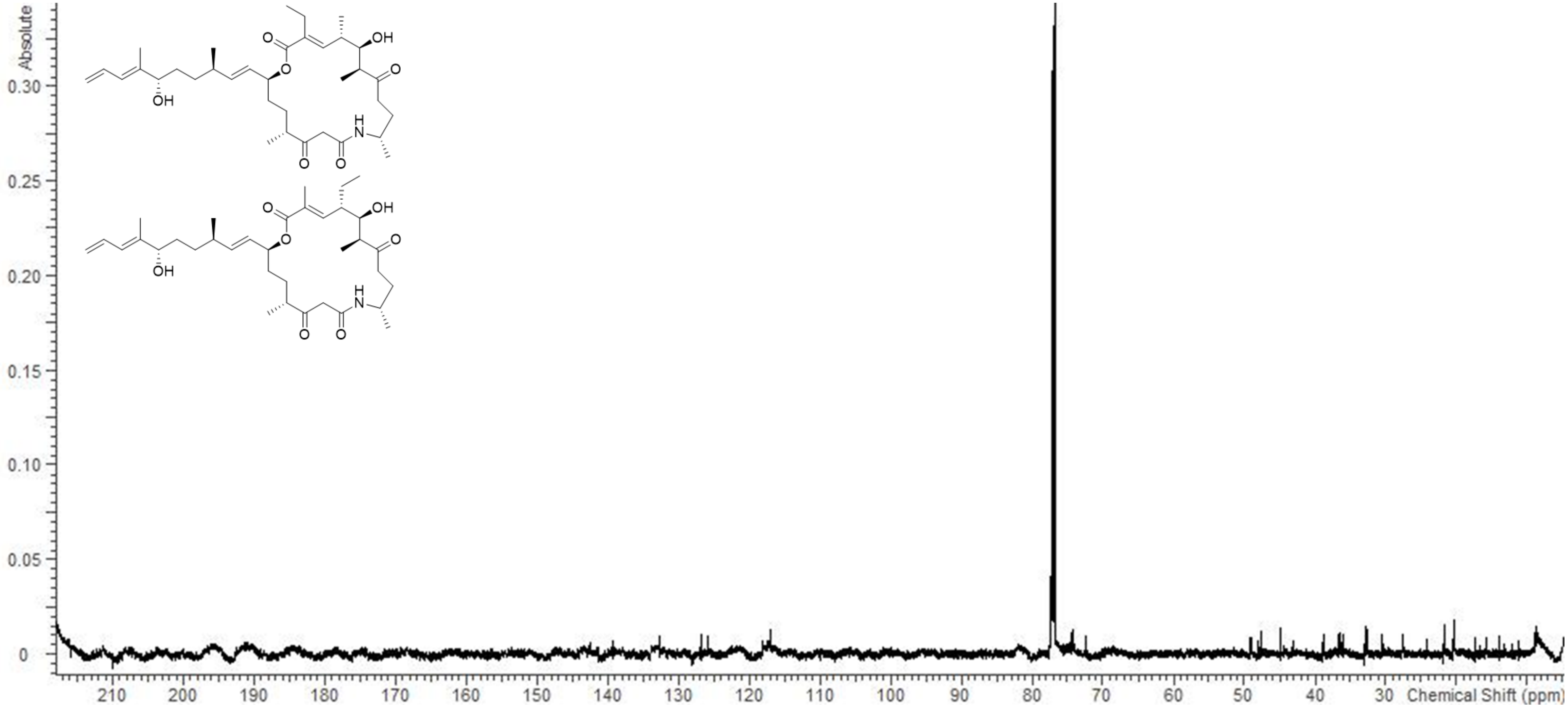

Figure S 31: ^{13}C spectrum of angiolam D$_1$ (4a) and D$_2$ (4b) in chloroform-*d* at 175 MHz.

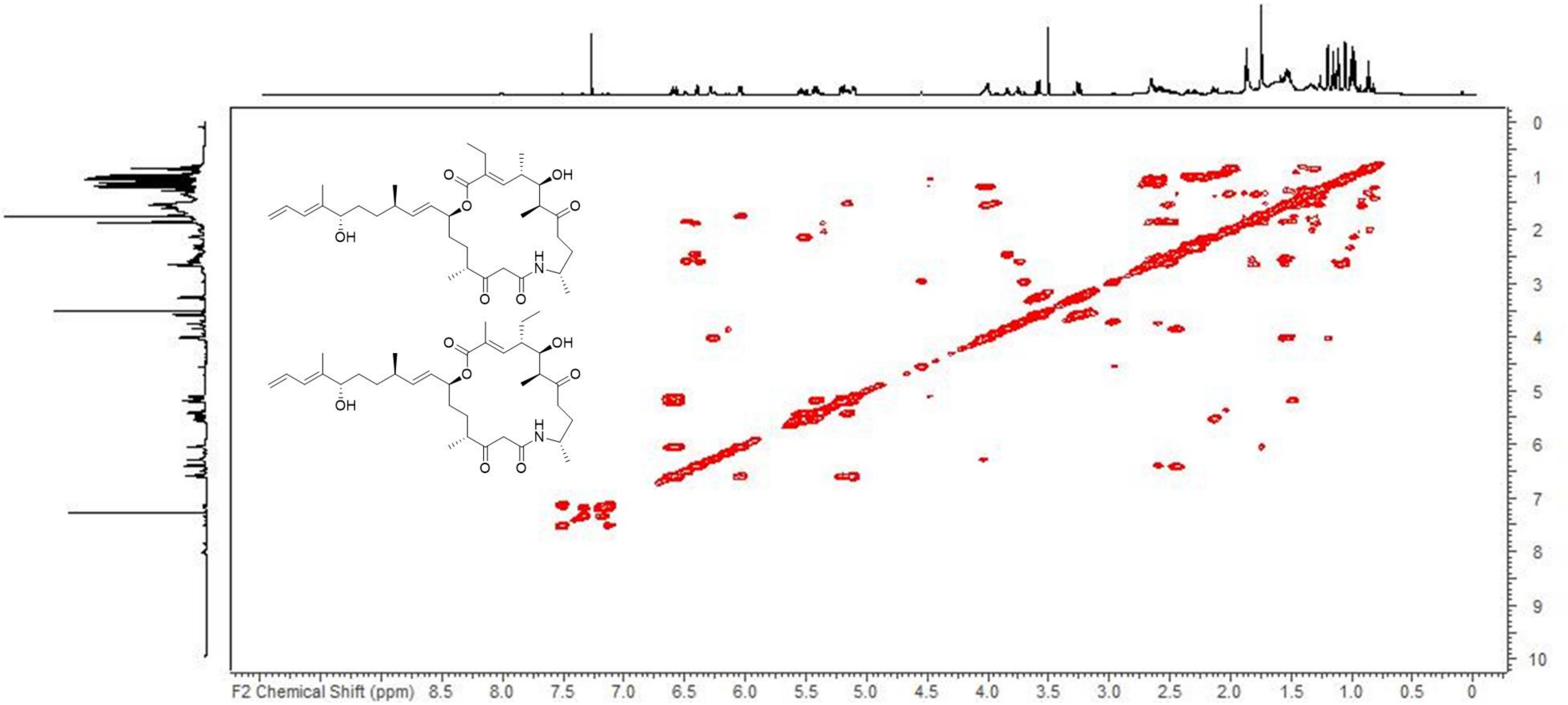

Figure S 32: COSY spectrum of angiolam D_1 (4a) and D_2 (4b) in chloroform-*d* at 700 MHz.

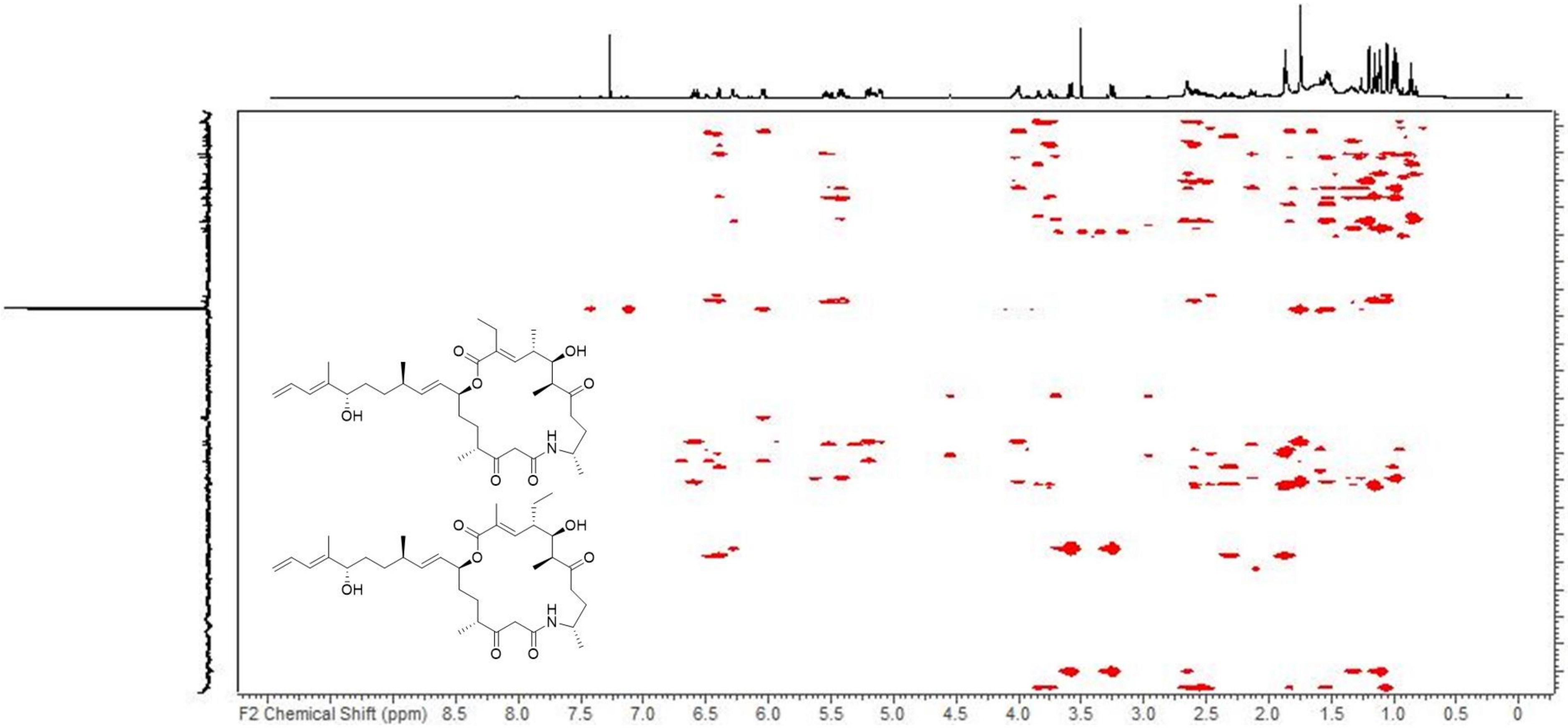

Figure S 33: HMBC spectrum of angiolam D$_1$ (4a) and D$_2$ (4b) in chloroform-*d* at 175/700 (F1/F2) MHz.

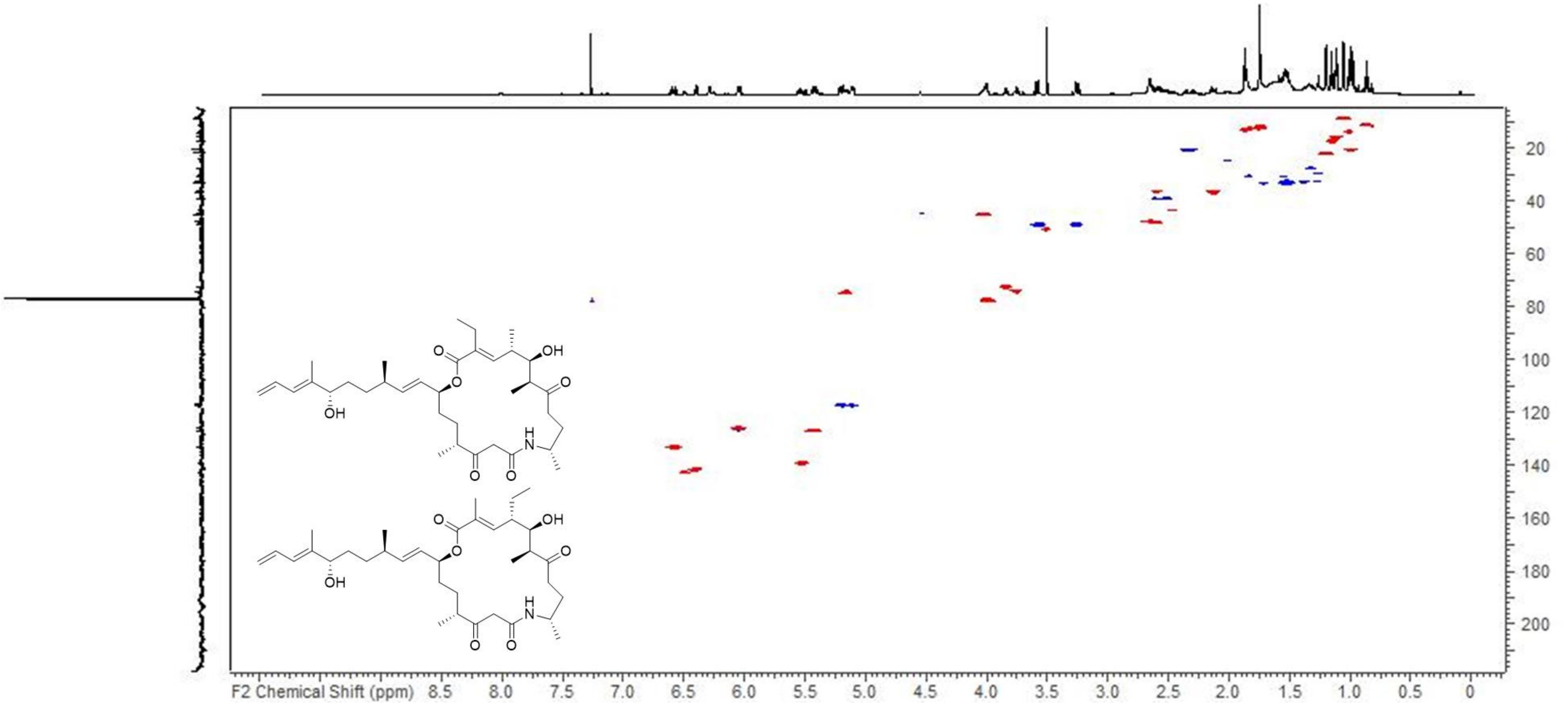

Figure S 34: HSQC spectrum of angiolam D_1 (4a) and D_2 (4b) in chloroform-*d* at 175/700 (F1/F2) MHz.

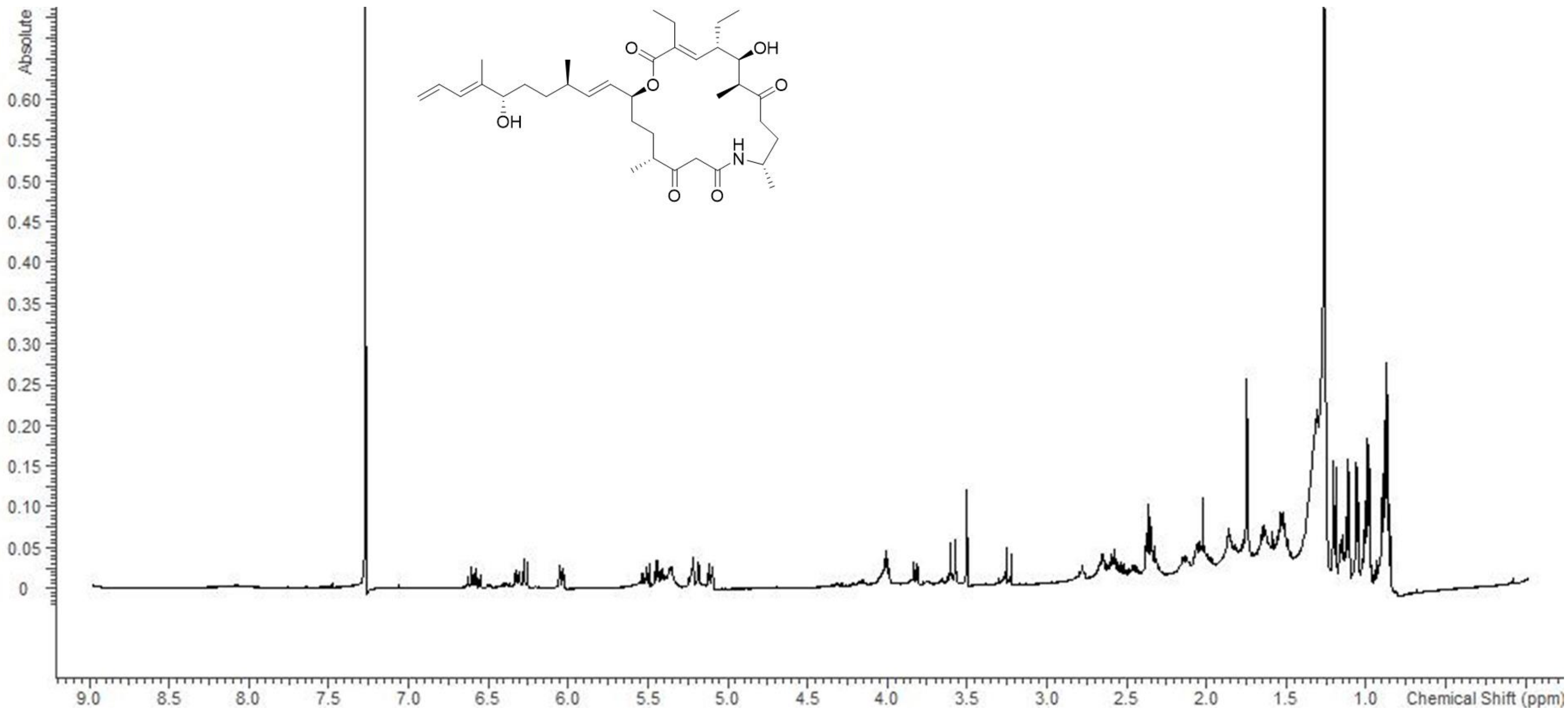

Figure S 35: ^{1}H spectrum of angiolam F (5) in chloroform-*d* at 500 MHz.

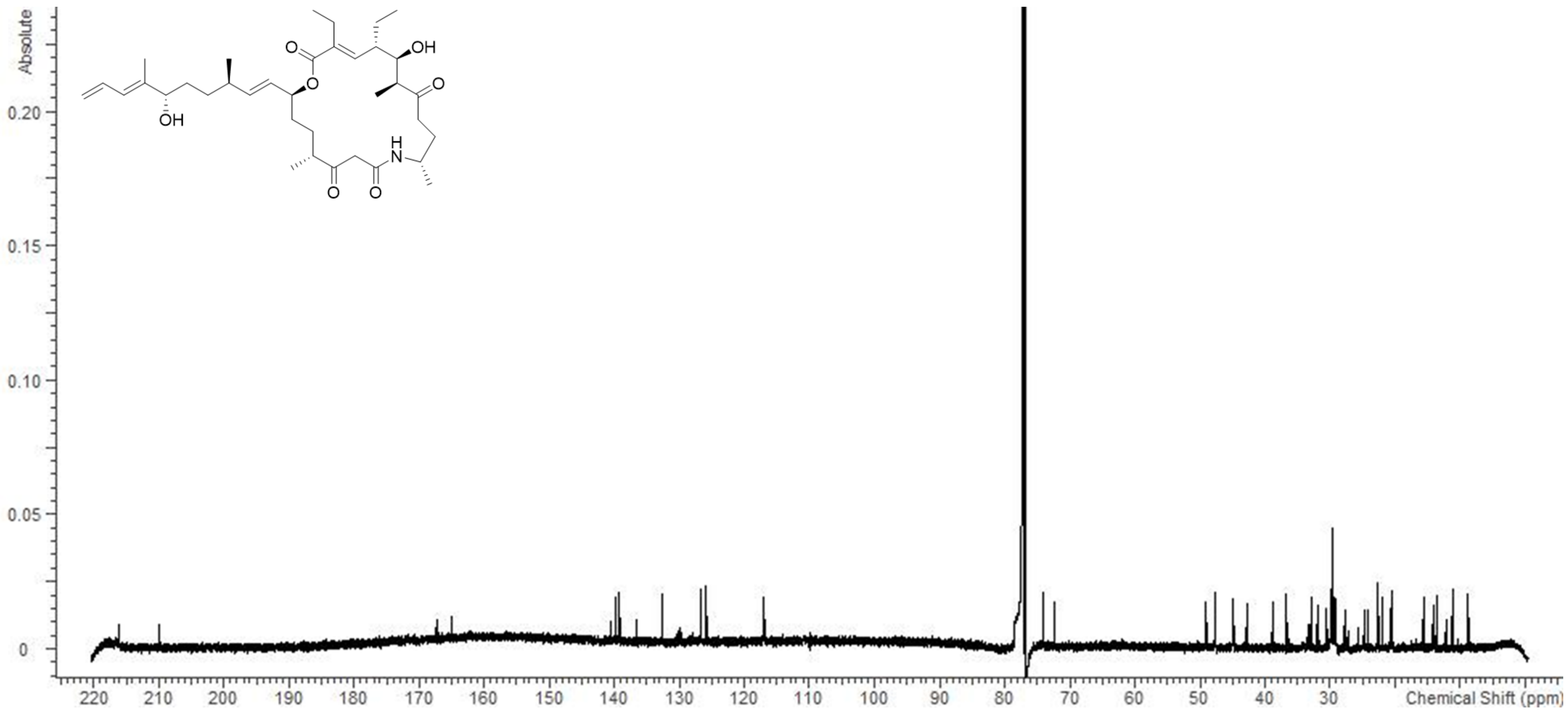

Figure S 36: ^{13}C spectrum of angiolam F (5) in chloroform-*d* at 125 MHz.

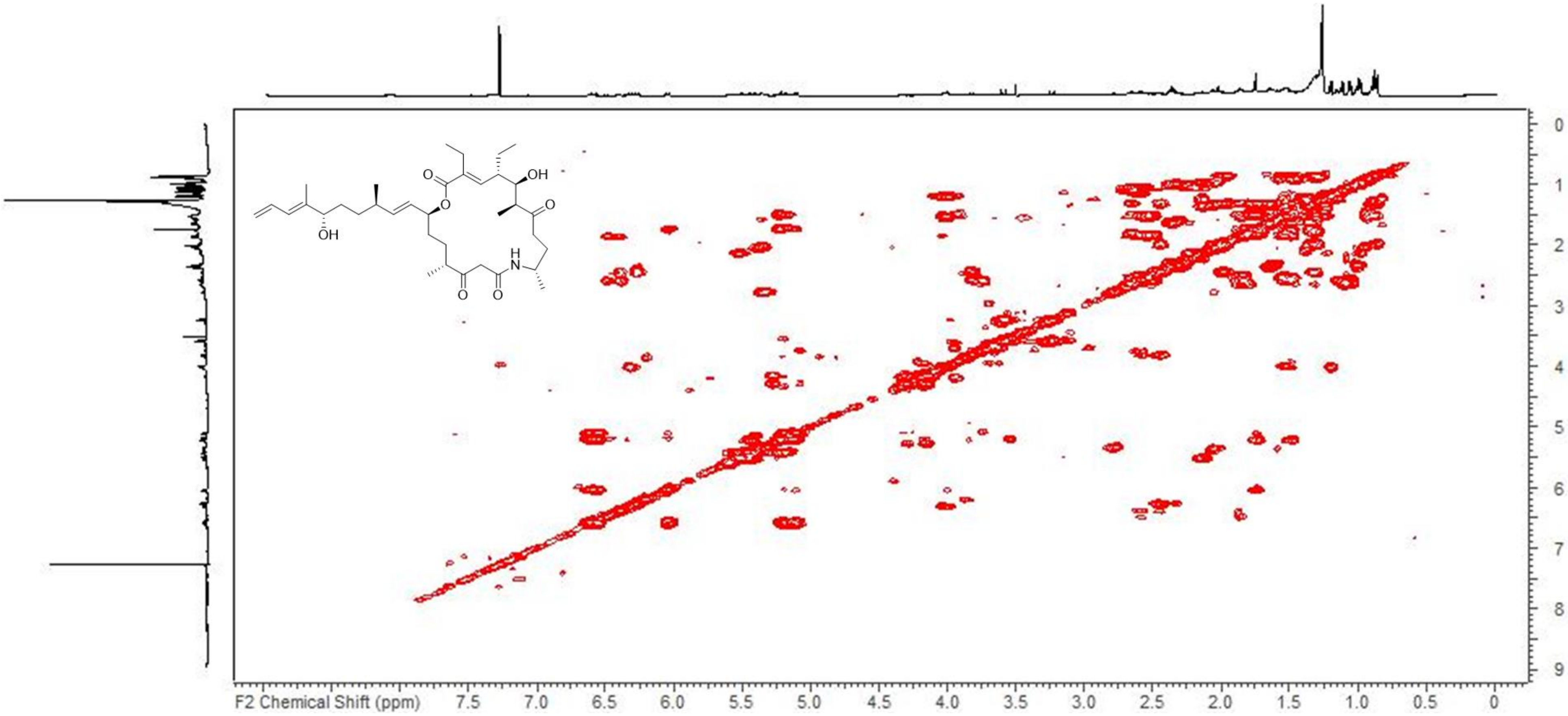

Figure S 37: COSY spectrum of angiolam F (5) in chloroform-*d* at 500 MHz.

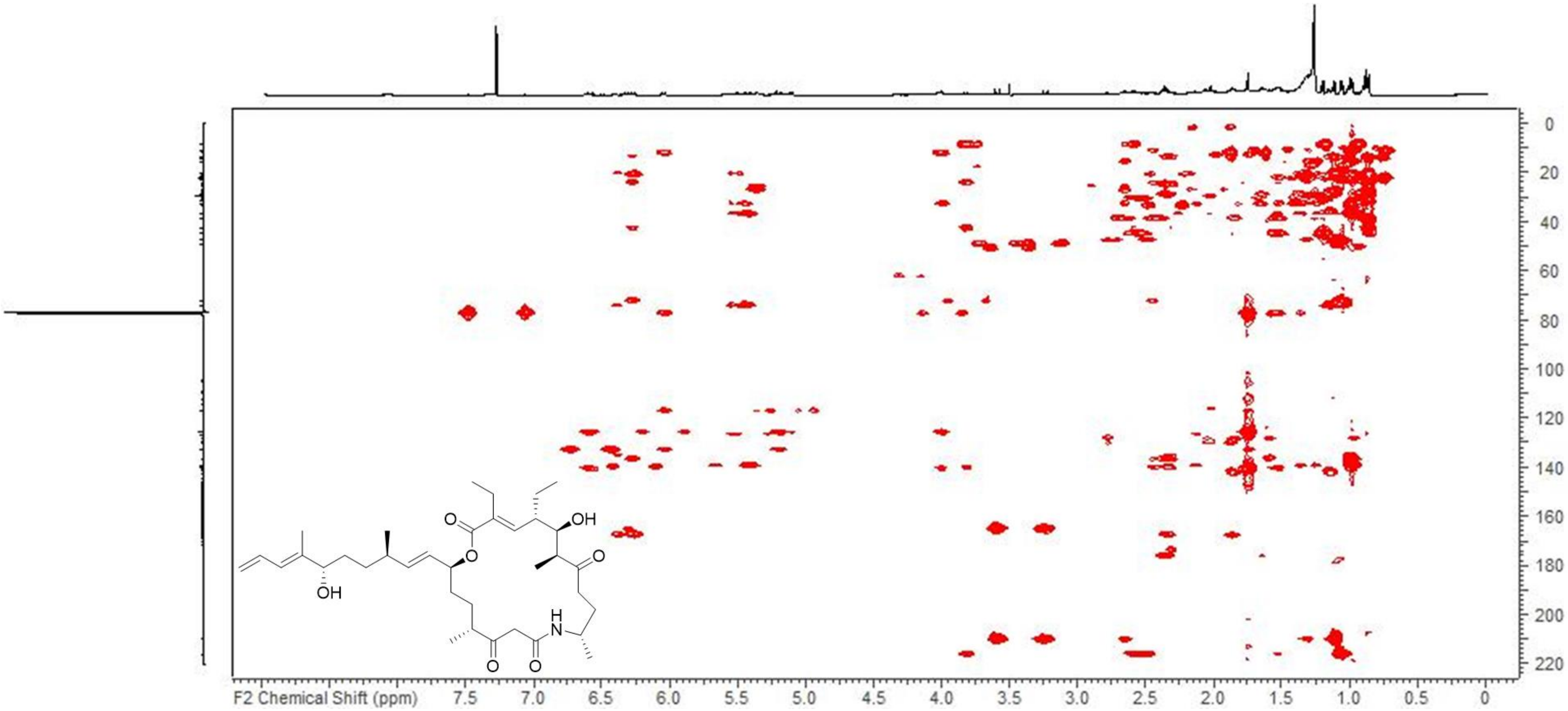

Figure S 38: HMBC spectrum of angiolam F (5) in chloroform-*d* at 125/500 (F1/F2) MHz.

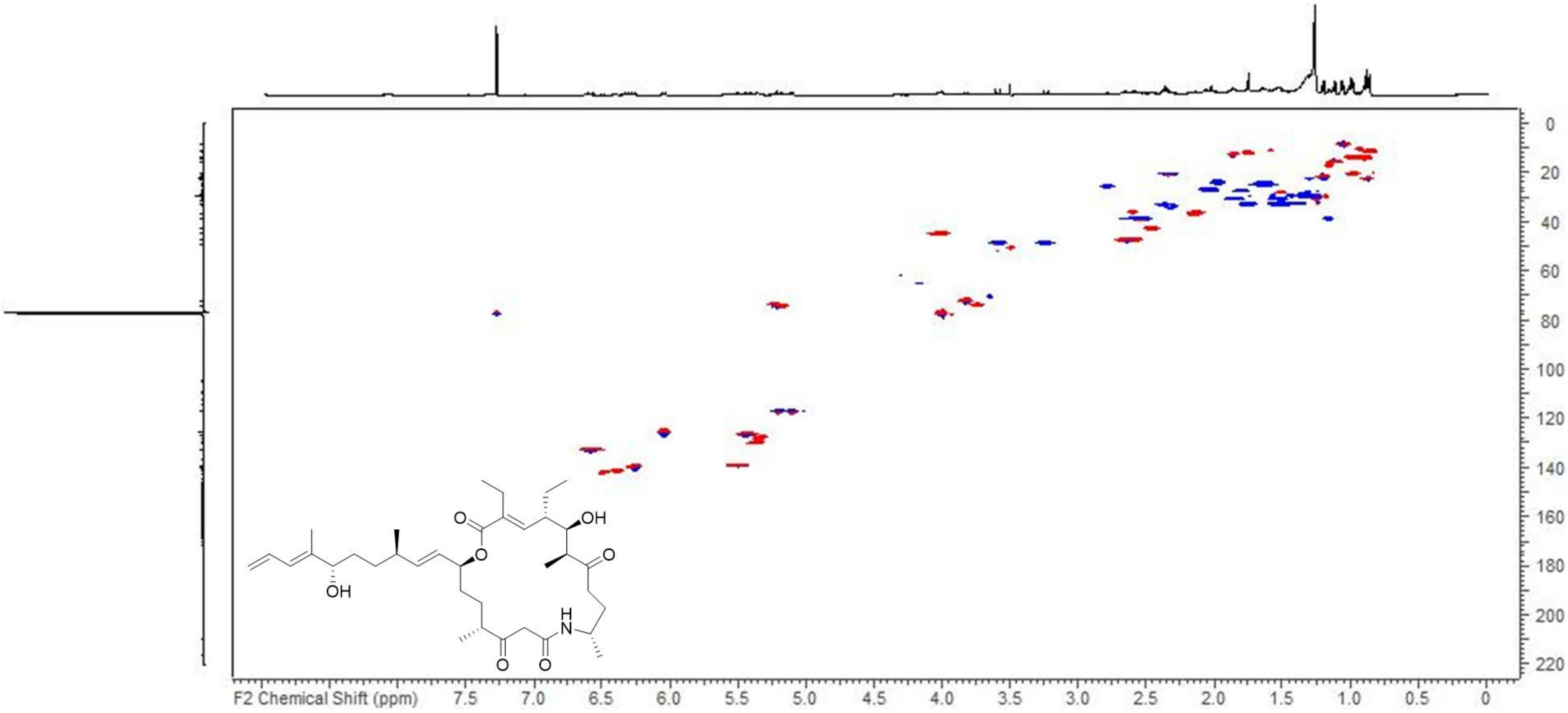

Figure S 39: HSQC spectrum of angiolam F (5) in chloroform-*d* at 125/500 (F1/F2) MHz.

4 References

1. Sandmann, A., Sasse, F. & Müller, R. Identification and analysis of the core biosynthetic machinery of tubulysin, a potent cytotoxin with potential anticancer activity. *Chem. Biol.* **11,** 1071–1079; 10.1016/j.chembiol.2004.05.014 (2004).

2. Nothias, L.-F. *et al.* Feature-based molecular networking in the GNPS analysis environment. *Nat Methods* **17,** 905–908; 10.1038/s41592-020-0933-6 (2020).

3. Wang, M. *et al.* Sharing and community curation of mass spectrometry data with Global Natural Products Social Molecular Networking. *Nat. Biotechnol.* **34,** 828–837; 10.1038/nbt.3597 (2016).

4. Werner Kohl, Barbara Witte, Brigitte Kunze, Victor Wray, Dietmar Schomburg, Hans Reichenbach und Gerhard Höfle. Angiolam A - ein neues Antibiotikum aus Angiococcus. *Liebigs Ann. Chem.,* 2088–2097 (1985).

5. Gieseler, M. T. & Kalesse, M. Synthesis of Angiolam A. *Org. Lett.* **16,** 548–551; 10.1021/ol403423r (2014).

6. Blin, K. *et al.* antiSMASH 6.0: improving cluster detection and comparison capabilities. *Nucleic Acids Res.* **49,** W29-W35; 10.1093/nar/gkab335 (2021).

7. Keatinge-Clay, A. T. The structures of type I polyketide synthases. *Nat. Prod. Rep.* **29,** 1050–1073; 10.1039/c2np20019h (2012).

8. Oliynyk, M. *et al.* Analysis of the biosynthetic gene cluster for the polyether antibiotic monensin in Streptomyces cinnamonensis and evidence for the role of monB and monC genes in oxidative cyclization. *Mol. Microbiol.* **49,** 1179–1190; 10.1046/j.1365-2958.2003.03571.x (2003).

9. Keatinge-Clay, A. T. A Tylosin Ketoreductase Reveals How Chirality is Determined in Polyketides. *Chem. Biol.* **14,** 898–908; 10.1016/j.chembiol.2007.07.009 (2007).

10. Kitsche, A. & Kalesse, M. Configurational assignment of secondary hydroxyl groups and methyl branches in polyketide natural products through bioinformatic analysis of the ketoreductase domain. *ChemBioChem* **14,** 851–861; 10.1002/cbic.201300063 (2013).

11. Kwan, D. H. *et al.* Prediction and manipulation of the stereochemistry of enoylreduction in modular polyketide synthases. *Chem. Biol.* **15,** 1231–1240; 10.1016/j.chembiol.2008.09.012 (2008).
